# Systematic Analysis of Zn_2_Cys_6_ Transcription Factors Required for Development and Pathogenicity by High-Throughput Gene Knockout in the Rice Blast Fungus

**DOI:** 10.1371/journal.ppat.1004432

**Published:** 2014-10-09

**Authors:** Jianping Lu, Huijuan Cao, Lilin Zhang, Pengyun Huang, Fucheng Lin

**Affiliations:** 1 School of Life Sciences Zhejiang University, Hangzhou, Zhejiang Province, China; 2 Biotechnology Institute, Zhejiang University, Hangzhou, Zhejiang Province, China; 3 China Tobacco Gene Research Center, Zhengzhou Tobacco Research Institute of CNTC, Zhengzhou, Henan Province, China; Purdue University, United States of America

## Abstract

Because of great challenges and workload in deleting genes on a large scale, the functions of most genes in pathogenic fungi are still unclear. In this study, we developed a high-throughput gene knockout system using a novel yeast-*Escherichia*-*Agrobacterium* shuttle vector, pKO1B, in the rice blast fungus *Magnaporthe oryzae*. Using this method, we deleted 104 fungal-specific Zn_2_Cys_6_ transcription factor (TF) genes in *M. oryzae*. We then analyzed the phenotypes of these mutants with regard to growth, asexual and infection-related development, pathogenesis, and 9 abiotic stresses. The resulting data provide new insights into how this rice pathogen of global significance regulates important traits in the infection cycle through Zn_2_Cys_6_TF genes. A large variation in biological functions of Zn_2_Cys_6_TF genes was observed under the conditions tested. Sixty-one of 104 Zn_2_Cys_6_ TF genes were found to be required for fungal development. In-depth analysis of TF genes revealed that TF genes involved in pathogenicity frequently tend to function in multiple development stages, and disclosed many highly conserved but unidentified functional TF genes of importance in the fungal kingdom. We further found that the virulence-required TF genes *GPF1* and *CNF2* have similar regulation mechanisms in the gene expression involved in pathogenicity. These experimental validations clearly demonstrated the value of a high-throughput gene knockout system in understanding the biological functions of genes on a genome scale in fungi, and provided a solid foundation for elucidating the gene expression network that regulates the development and pathogenicity of *M. oryzae*.

## Author Summary

*Magnaporthe oryzae* is not only the fungus causing the rice blast disease, which leads to 20–30% losses in rice production, but also a primary model pathosystem for understanding host-pathogen interactions. However, there is no high-throughput gene knockout system constructed, and little is known about most of the genes in this fungus. We developed a high-throughput gene knockout system, and using this system, we obtained null mutants of 104 fungal-specific Zn_2_Cys_6_ transcription factor (TF) genes by screening 8741 primary transformants in *M. oryzae*. We analyzed the functions of these TF genes in development, pathogenesis, and stress responses under 9 conditions. We found that 61 Zn_2_Cys_6_ TF genes play indispensable and diversified roles in fungal development and pathogenicity. *CNF1* is the first reported TF gene that strongly and negatively regulates asexual development in the rice blast fungus, and *CCA1*, *CNF1*, *CNF2*, *CONx1, GPF1*, *GTA1*, *MoCOD1* and *PCF1* are required for pathogenicity. We further found via RNA-seq that *GPF1* and *CNF2* have similar mechanisms in gene expression regulation related to pathogenicity. The resulting data provide new insights into how Zn_2_Cys_6_ TF genes regulate important traits during the infection cycle of this rice blast pathogen.

## Introduction

*Magnaporthe oryzae* is the best-studied phytopathogenic fungus, which was voted first in the top 10 list of fungal plant pathogens by an international community of molecular plant pathologists [1]. The importance of this filamentous ascomycete fungus is not only owing to the fact that the rice blast disease caused by the fungus is the most destructive disease of rice throughout the world, which typically leads to 20–30% losses and even complete loss in grain production during regional epidemics [1], [2], but also to its being a primary model in the study of host–fungal pathogen interactions [3]. The rice blast fungus has a complicated life cycle including hyphal growth, conidiogenesis, conidial germination, appressorium formation and plant infection, which provides substantial biological information in eukaryotic development and pathogenesis. The rice blast fungus is highly amenable to molecular genetic manipulation, and the functions of numerous genes are identified by gene knockout or ectopic insertion [4]–[6].

Transcription factors (TFs) are proteins that bind to specific DNA sequences, thereby controlling the flow of genetic information from DNA to mRNA. In the rice blast fungus, more than 522 putative TF proteins have been identified from 12,991 *M. oryzae* proteins (www.ftfd.snu.ac.kr; www.broadinstitute.org). Therefore, nearly 4.02% of genes in the genome code for TFs, which makes this family the single largest family of *M. oryzae* proteins. Recent functional analyses of single or several TF genes revealed their critical biological roles in fungal development, pathogenesis and response to the environment, for instance, in hyphal growth (*MNH6*, *MSTU1*, *MoCRZ1* and *MoSWI6*) [7]–[10], conidiogenesis (*COM1*, *CON7*, *COS1*, *MNH6*, and *MoHOX2*/*HTF1*) [7], [11]–[16], conidial germination (*TRA1*) [17], appressorium formation (*MoLDB1*, *MoSOM1* and *MoCDTF1*) [18], [19], plant infection (*COM1*, *MNH6*, *MIG1*, *MST12*/*MoHOX8*, and *MoSfl1*) [7], [11], [16], [20]–[22], and response to oxidative stress(*MoATF1* and *MoAP1*) [23], [24] or light (*MgWC-1*) [25]. However, the biological functions of most TFs have not been revealed, mainly because it is difficult to delete genes on a large scale and because a high-throughput gene knockout method has not been previously established in *M. oryzae*.

Several loss-of-function techniques, such as homologous recombination [4], insertional mutagenesis [6] and RNA interference [26], have been used to investigate gene functions in fungi. Although insertional mutagenesis and RNA interference have been effectively used to inactivate gene functions, they all have limitations that prevent them from becoming high-throughput gene knockout techniques, including 1) inability to cover the genome and the heavy workload to clone genes in the insertional mutagenesis method [6], and 2) the silencing of potential unintended targets by the RNA interference method [26]. Gene knockout through DNA homologous recombination is a primary technique used to inactivate gene functions. More than 650 putative TF genes were disrupted in *Fusarium graminearum* using homologous recombination [27]. In that study, the double-joint PCR method [28] was used to generate gene knockout constructs, which were then transformed into fungal protoplasts. In *Neurospora crassa*, homologous recombination was developed into a high-throughput gene knockout technique [29], in which gene knockout constructs generated by making use of a yeast recombinational cloning method were transformed into conidia by electroporation. Yeast recombinational cloning is a more suitable method than double-joint PCR for high-throughput gene knockout study. However, protoplast transformation is an elaborate, time-consuming and inefficient method, and electroporation of germinating conidia has not been built in the rice blast fungus and many other filamentous fungi. The frequency of homologous recombination after transformation is often quite low in filamentous fungi, typically <10% in *M. oryzae* [30]. In addition, mutant screening is a tedious and inefficient step in high-throughput gene knockout studies. The implementation of *KU70/KU80* (*mus-51/mus-52*) mutations as gene knockout background strains greatly increases the frequency of homologous recombination in *N. crassa*, Aspergilli and other filamentous fungi, and reduces the workload in screening [29], [31]–[34]. However, the null mutants obtained from *KU70/KU80* deletion background strains need to be complemented with native *KU70/KU80* genes before analyzing their mutant phenotypes [29]. These shortcomings limit the application of the above methods in research in fungal functional genomics. Until now, there is still a surge in interest in functional genomics research through the systematic mutagenesis of identified genes sequenced in the genomes of a large number of fungi (http://www.ncbi.nlm.nih.gov/genome/).

To learn the biological functions of TFs at the genome level, we constructed a high-throughput gene knockout method that enables the rapid knockout of large numbers of genes in the rice blast fungus. In this method, gene knockout vectors are built by a yeast recombinational cloning method using a high-throughput way, DNA transformations are performed by *Agrobacterium tumefaciens*-mediated transformation (ATMT), and the null mutants are identified by the negative (*GFP*)/positive (a resistant gene) screening system through a novel yeast-*Escherichia*-*Agrobacterium* shuttle vector, pKO1B. With this system, we deleted 104 putative fungal-specific Zn_2_Cys_6_ TF genes in *M. oryzae*. The null mutants were then examined for their phenotypes in development, pathogenicity and responses to stress conditions. In particular, many Zn_2_Cys_6_ TF genes required for fungal growth, asexual development, conidial germination and appressorium formation, pathogenicity, and response to stress were identified. We further identified the genes regulated by *GPF1* and *CNF2* via RNA-sequencing (RNA-seq) and found that *GPF1* and *CNF2* have similar mechanisms in the regulation of gene expression related to fungal pathogenicity. Our findings will provide new insights into the transcriptional regulation of fungal development and pathogenicity, and a way to study the biological functions of genes in fungi at the genome level.

## Results

### Strategy of the high-throughput gene knockout system via pKO1B vector in *M. oryzae*

**Figure 1:**
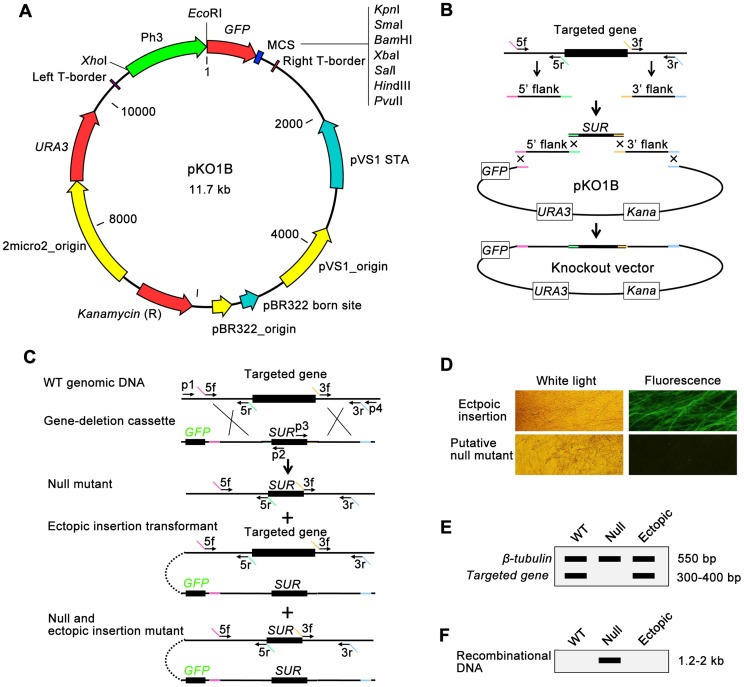
Overview of the high-throughput gene knockout system in fungus. (**A**) Features of a new binary yeast-*Escherichia*-*Agrobacterium* shuttle vector, pKO1B. (**B**) Building of gene-deletion cassettes in pKO1B by yeast recombinational cloning. The 5′ and 3′ flanking fragments of the targeted genes were separately amplified from genomic DNA with primers 5f/5r and 3f/3r. Primers 5r and 3f have 5′ tails homologous to the *SUR* cassette, whereas those for 5f and 3r are homologous to the vector. The two flanks were cotransformed into yeast along with the *SUR* cassette and gapped pKO1B. Homologous recombination created the circular knockout vector, and the final knockout vector was subsequently transformed into *A. tumefaciens*. (**C**) Deletion of the targeted gene. The gene-deletion cassette was transformed into the fungal cells via ATMT. Homologous recombination created three types of transformants: null mutants, ectopic insertion transformants, and null and ectopic insertion mutants. The *GFP* gene was discarded in the null mutants. Primers p1/p2 or p3/p4 were used to identify the unique recombinational DNA fragment, indicating a knockout event. (**D**) The transformants are screened for GFP fluorescence under a microscope. Putative null mutants do have not GFP fluorescence, but ectopic transformants do. (**E**) The transformants are screened by double PCR for the targeted gene using the *β-tubulin* gene as a positive control. The wild-type strain or ectopic transformants produced a characteristic band, indicating the targeted gene, while the null mutants did not. (**F**) The transformants are screened by PCR for a unique recombinational DNA fragment marked as a knockout event. The null mutants have a 1.2–2.0 kb band on an electrophoretic gel, while the wild-type strain and the ectopic transformants do not.

Three major challenges in high-throughput gene knockout of fungi are 1) how to build gene-deletion cassettes quickly, 2) how to transfer DNA into fungal cells easily, and 3) how to identify null mutants from the large number of transformants efficiently. To solve these problems, we developed a high-throughput gene knockout system using a newly designed yeast-*Escherichia*-*Agrobacterium* shuttle vector, pKO1B, (Figure 1A). Through this plasmid, three techniques suitable for high-throughput manipulation (yeast recombinational cloning to build gene-deletion cassettes, ATMT to transfer DNA into fungal cells and dual selection system to identify null mutants) were combined into the high-throughput gene knockout system.

pKO1B is a vector built on the framework of the binary vector pCAMBIA1300 (www.cambia.org). It contains the *URA3*-2micro2_origin sequence from a yeast plasmid pYES2 (Invitrogen, USA) and *eGFP* gene under the control of a strong promoter of *M. oryzae H3* histone gene [35] (Figure 1A). pKO1B has a new characteristic: the ability to be replicated in three organisms, *Saccharomyces cerevisiae*, *Escherichia coli* and *A. tumefaciens*. Through the gapped vector pKO1B, three DNA fragments (5′ and 3′ flanking fragments of the targeted gene and a resistant gene fragment) could be merged into a gene-deletion cassette in one step by yeast recombinational cloning (Figure 1B). The knockout vectors obtained were transformed into *A. tumefaciens* without transferring the gene-deletion cassettes from a yeast plasmid to another binary *Agrobacterium* plasmid. The gene-deletion cassettes in pKO1B were transformed into fungal cells using the ATMT method. When the gene-deletion cassettes were ectopically integrated into fungal genomic DNA, *GFP* was activated in ectopic insertional transformants and was easily observed under a fluorescence microscope as a negative selective marker, but not in null mutants (Figure 1C and 1D).

To screen null mutants efficiently, the genomic DNAs of the transformants without green fluorescence were isolated using an improved CTAB method in a high-throughput way (shown in Materials and Methods) and then were detected for the targeted gene and *β-tubulin* gene by double PCR. If the targeted gene was deleted in a mutant, only one band for *β-tubulin* appeared as a positive control on an electrophoretic gel; otherwise, there were two bands with one for the targeted gene and the other for *β-tubulin* in ectopic insertion transformants (Figure 1C and 1E). For those null mutants identified in the above negative screening PCR, we then continued to search for the unique recombinational DNA fragment that indicated a knockout event and only appeared in the null mutants by PCR. In this PCR, one primer was limited in the genomic DNA outside of the 5′ or 3′ flanking fragment of the targeted gene, and the other primer was limited in the resistant gene of the gene-deletion cassettes (primers p1+p2 or p3+p4 in Figure 1C). There was one band 1.2–2.0 kb in length on the electrophoretic gel that appeared in null mutants. Otherwise, there was no band appearing in the ectopic transformants and wild-type strain (Figure 1F). Gene knockout and ectopic insertion maybe happened coincidentally (Figure 1C). To identify those mutants that were undetected in previous screening steps, copies of transformed gene deletion cassettes in null mutants were confirmed by qPCR after comparison with the wild-type strain using *β-tubulin* as a control. The null mutants had one copy of the gene deletion cassette, while the null+ectopic transformants had more than two copies and the wild-type strain none. Finally, the mutants containing a single copy of the gene deletion cassette were considered null mutants.

TFs containing fungal-specific Zn_2_Cys_6_ zinc finger and fungal_TF_MHR Domain are the largest group of TFs in the rice blast fungus. The 163 putative fungal-specific Zn_2_Cys_6_ genes, previously annotated at the Fungal Transcription Factor Database (http://ftfd.snu.ac.kr/index.php) [36] or obtained by BLAST searches at NCBI under organism item (rice blast fungus, taxid: 318829) with the Conserved Domain program [37], were selected to generate gene deletion mutants (Table S1 in Text S1). As a result, 104 TF genes were identified to be deleted in *M. oryzae* (Table S2 and Table S3 in Text S1). The knockout rate of 104 genes was 15.03±12.02% (from 0.68 to 62.07%). The causes for tens of TF genes not deleted in this experiment lie in the following. 1) Knockout vectors of twenty-one genes had not been constructed. 2) For those genes with a knockout rate less than 1%, more transformants need to be screened. 3) Because the *SUR* gene was selected as a resistance gene, the mutants must be screened on DCM medium. But the mutants of TF genes that are involved with amino acid metabolism cannot grow on DCM medium. 4) Some genes may be essential genes.

### Phenotypic analyses of fungal-specific Zn_2_Cys_6_ transcription factor deletion mutants at developmental stages

**Figure 2:**
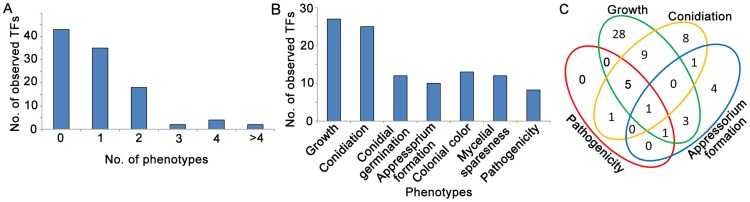
Analysis of Zn_2_Cys_6_ transcription factor mutant phenotypes in fungal development stages. (**A**) Number of TFs showing multiple mutant phenotypes. (**B**) Number of mutants showing mutant phenotypes in each developmental stage. (**C**) Venn diagram showing the number of mutant phenotypes. The phenotypes included vegetative growth (conidial germination, colony growth, pigmentation and mycelial appearance), conidiation (asexual reproduction), appressorium formation and pathogenicity to rice and barley.

We analyzed the phenotypes of the 104 Zn_2_Cys_6_ TF null mutants at multiple developmental stages that *M. oryzae* likely encounters during its infection cycle in rice. These phenotypes analyzed included: developmental characteristics (mycelial growth, colony color and mycelial shape; conidiation; conidial germination; and appressorium formation) and virulence to plants (barley leaf explants, rice leaf explants and rice seedlings). A substantial fraction of the mutants (58.7%, 61/104) clearly displayed visible phenotypes (Figure 2A). The results (Figure 2B) revealed that 27 of the 104 TF genes studied were involved in mycelial growth (Figure S1), 25 TF genes in conidial production, 12 TF genes in conidial germination and 10 TF genes in appressorium formation, and 5 TF genes were involved in pathogenicity in barley (Figure S2), while 7 TF genes were involved in pathogenicity in rice (Figure S3). Table 1 and Table S4 in Text S1 provide a complete list of the phenotypic analyses. Among these mutants displaying clearly visible mutant phenotype changes, 42.6% (26/61) of mutants exhibited multiple mutant phenotypes and 57.4% (35/61) exhibited a single mutant phenotype (Figure 2A). The Zn_2_Cys_6_ TFs regulating asexual reproduction also often controlled vegetative growth (conidial germination, colony growth, pigmentation and mycelial appearance) of the fungus (Figure 2C). Interestingly, 7 TF genes required for pathogenicity had multiple mutant phenotypes (Figure 2C, Table 1), and this phenomenon implied that the fungal pathogenic process is complex, where it is closely linked with many fungal development stages, and that the pathogenicity-related Zn_2_Cys_6_ TF genes are also involved in other developmental processes.

**Table 1:** Phenotypic summary of 104 Zn_2_Cys_6_ TF gene-deleted mutants.

Gene locus (MG8)	Gene	Growth	Conidiation	Conidial germination	Appressorium formation	Virulence	Stress
MGG_04387	*FZC1*	Normal	Normal	Normal	Normal	Normal	Affected
MGG_01486	*FZC2*	Normal	Normal	Normal	Reduced	Normal	Affected
MGG_01518	*MoNIT4*	Normal	Reduced	Normal	Normal	Normal	Affected
MGG_01624	*FZC3*	Normal	Normal	Normal	Normal	Normal	Unaffected
MGG_01734	*FZC4*	Normal	Normal	Normal	Normal	Normal	Affected
MGG_01833	*FZC5*	Normal	Reduced	Normal	Normal	Normal	Affected
MGG_02377	*FZC6*	Normal	Normal	Normal	Normal	Normal	Unaffected
MGG_02595	*FZC7*	Normal	Normal	Normal	Reduced	Normal	Unaffected
MGG_02879	*FZC8*	Reduced	Normal	Normal	Normal	Normal	Unaffected
MGG_02880	*FZC9*	Normal	Normal	Normal	Normal	Normal	Unaffected
MGG_03463	*FZC10*	Normal	Normal	Normal	Normal	Normal	Affected
MGG_04108	*TAS1*	Normal	Normal	Normal	Normal	Normal	Affected
MGG_04843	*FZC11*	Normal	Normal	Normal	Normal	Normal	Unaffected
MGG_05829	*FZC12*	Normal	Normal	Normal	Normal	Normal	Affected
MGG_05891	*FZC13*	Reduced	Reduced	Normal	Normal	Normal	Affected
MGG_06355	*FZC14*	Normal	Reduced	Normal	Normal	Normal	Affected
MGG_07063	*GCC1*	Reduced	None			Normal	Affected
MGG_07800	*FZC15*	Normal	Normal	Normal	Normal	Normal	Affected
MGG_08094	*FZC16*	Normal	Normal	Reduced	Normal	Normal	Affected
MGG_08130	*FZC17*	Normal	Normal	Normal	Normal	Normal	Affected
MGG_08777	*FZC18*	Normal	Normal	Normal	Normal	Normal	Affected
MGG_10197	*TRA1*	Normal	Normal	Reduced	Normal	Normal	Unaffected
MGG_11116	*FZC19*	Normal	Normal	Normal	Normal	Normal	Affected
MGG_12037	*FZC20*	Normal	Normal	Normal	Normal	Normal	Affected
MGG_12424	*FZC21*	Reduced	Normal	Normal	Normal	Normal	Affected
MGG_16444	*FZC22*	Normal	Reduced	Normal	Normal	Normal	Affected
MGG_16756	*FZC23*	Normal	Normal	Normal	Normal	Normal	Unaffected
MGG_17060	*FZC24*	Normal	Normal	Normal	Normal	Normal	Affected
MGG_17669	*FZC25*	Normal	Reduced	Normal	Normal	Normal	Unaffected
MGG_17821	*FZC26*	Normal	Reduced	Normal	Normal	Normal	Affected
MGG_17841	*GPF1*	Reduced	Normal	Reduced	Reduced	None	Affected
MGG_08058	*MoACEII*	Reduced	Normal	Normal	Normal	Normal	Affected
MGG_00329	*FZC27*	Normal	Normal	Normal	Reduced	Normal	Affected
MGG_07681	*FZC28*	Reduced	Normal	Normal	Normal	Normal	Affected
MGG_01779	*FZC29*	Normal	Normal	Normal	Normal	Normal	Affected
MGG_01887	*FZC30*	Normal	Normal	Normal	Normal	Normal	Affected
MGG_02866	*FZC31*	Reduced	Normal	Normal	Normal	Normal	Affected
MGG_04674	*FZC32*	Normal	Normal	Normal	Normal	Normal	Affected
MGG_05033	*FZC33*	Reduced	Normal	Reduced	Normal	Normal	Affected
MGG_05343	*MoCOD1*	Reduced	None			Reduced	Affected
MGG_06279	*FZC34*	Normal	Normal	Normal	Normal	Normal	Affected
MGG_06312	*FZC35*	Normal	Normal	Normal	Normal	Normal	Unaffected
MGG_06416	*FZC36*	Normal	Normal	Normal	Normal	Normal	Unaffected
MGG_06626	*FZC37*	Normal	Reduced	Normal	Normal	Normal	Affected
MGG_06778	*FZC38*	Normal	Normal	Normal	Normal	Normal	Unaffected
MGG_06832	*FZC39*	Normal	Normal	Normal	Normal	Normal	Affected
MGG_06954	*FZC40*	Normal	Normal	Normal	Normal	Normal	Unaffected
MGG_07149	*GTA1*	Reduced	Reduced	Reduced	Normal	Reduced	Affected
MGG_07215	*PIG1*	Normal	Normal	Normal	Normal	Normal	Affected
MGG_07534	*FZC41*	Normal	Normal	Normal	Normal	Normal	Affected
MGG_07549	*FZC42*	Reduced	Normal	Normal	Normal	Normal	Affected
MGG_07777	*FZC43*	Normal	Reduced	Reduced	Normal	Normal	Affected
MGG_07845	*FZC44*	Increased	Normal	Normal	Normal	Normal	Unaffected
MGG_08185	*FZC45*	Increased	Reduced	Normal	Normal	Normal	Affected
MGG_08314	*FZC46*	Reduced	Normal	Normal	Normal	Normal	Unaffected
MGG_08784	*FZC47*	Normal	Normal	Normal	Normal	Normal	Affected
MGG_14728	*FZC48*	Normal	Normal	Normal	Normal	Normal	Unaffected
MGG_09027	*FZC49*	Normal	Normal	Normal	Normal	Normal	Affected
MGG_09273	*FZC50*	Reduced	Normal	Reduced	Normal	Normal	Affected
MGG_09312	*FZC51*	Normal	Normal	Normal	Normal	Normal	Affected
MGG_09676	*FZC52*	Normal	Normal	Normal	Normal	Normal	Affected
MGG_09829	*FZC53*	Normal	Normal	Normal	Normal	Normal	Affected
MGG_09950	*FZC54*	Reduced	Normal	Normal	Normal	Normal	Affected
MGG_11764	*FZC55*	Normal	Normal	Normal	Reduced	Normal	Affected
MGG_12776	*FZC56*	Normal	Reduced	Normal	Normal	Normal	Affected
MGG_13350	*FZC57*	Normal	Reduced	Normal	Normal	Normal	Affected
MGG_13360	*CNF3*	Normal	Increased	Normal	Normal	Normal	Unaffected
MGG_13385	*FZC58*	Normal	Normal	Normal	Normal	Normal	Affected
MGG_13629	*FZC59*	Normal	Normal	Normal	Normal	Normal	Affected
MGG_14175	*FZC60*	Normal	Normal	Normal	Normal	Normal	Affected
MGG_14852	*FZC61*	Normal	Reduced	Normal	Normal	Normal	Affected
MGG_15023	*CNF2*	Normal	Increased	Normal	Normal	Reduced	Affected
MGG_17623	*PCF1*	Normal	Reduced	Reduced	Normal	Reduced	Affected
MGG_00021	*FZC62*	Increased	Reduced	Normal	Normal	Normal	Affected
MGG_00096	*FZC63*	Increased	Normal	Normal	Normal	Normal	Affected
MGG_00320	*FZC64*	Normal	Normal	Normal	Normal	Normal	Affected
MGG_00417	*FZC65*	Normal	Normal	Normal	Normal	Normal	Affected
MGG_00494	*MoPRO1*	Normal	Reduced	Normal	Reduced	Normal	Unaffected
MGG_02089	*FZC66*	Normal	Normal	Normal	Normal	Normal	Affected
MGG_02289	*FZC67*	Increased	Normal	Normal	Normal	Normal	Affected
MGG_02962	*CNF1*	Normal	Increased	Reduced	Normal	Reduced	Affected
MGG_03183	*CNF4*	Normal	Increased	Normal	Normal	Normal	Affected
MGG_03669	*FZC68*	Normal	Normal	Normal	Normal	Normal	Affected
MGG_04141	*FZC69*	Reduced	Normal	Normal	Reduced	Normal	Affected
MGG_04326	*FZC70*	Reduced	Normal	Normal	Normal	Normal	Affected
MGG_04360	*FZC71*	Normal	Normal	Normal	Normal	Normal	Affected
MGG_04933	*FZC72*	Normal	Normal	Normal	Normal	Normal	Affected
MGG_05153	*FZC73*	Reduced	Normal	Normal	Normal	Normal	Affected
MGG_05659	*CCA1*	Reduced	Reduced	Reduced	Reduced	Reduced	Affected
MGG_05724	*FZC74*	Normal	Normal	Normal	Normal	Normal	Affected
MGG_05845	*FZC75*	Normal	Normal	Normal	Reduced	Normal	Affected
MGG_06243	*FZC76*	Normal	Normal	Normal	Normal	Normal	Affected
MGG_06455	*FZC77*	Normal	Normal	Normal	Normal	Normal	Affected
MGG_07458	*FZC78*	Normal	Normal	Normal	Normal	Normal	Unaffected
MGG_07636	*FZC79*	Increased	Normal	Normal	Normal	Normal	Affected
MGG_08361	*FZC80*	Normal	Normal	Normal	Normal	Normal	Affected
MGG_08974	*FZC81*	Normal	Normal	Normal	Normal	Normal	Affected
MGG_12339	*FZC82*	Normal	Normal	Reduced	Reduced	Normal	Affected
MGG_12349	*CONx1*	Normal	Reduced			Reduced	Affected
MGG_13927	*FZC83*	Increased	Normal	Normal	Normal	Normal	Affected
MGG_14816	*FZC84*	Normal	Normal	Reduced	Normal	Normal	Affected
MGG_15139	*FZC85*	Normal	Normal	Normal	Normal	Normal	Affected
MGG_17012	*FZC86*	Normal	Normal	Normal	Normal	Normal	Affected
MGG_03711	*FZC87*	Reduced	Normal	Normal	Normal	Normal	Affected

Notes: The phenotypes of the mutants in colony growth, conidiation, conidial germination, appressorium formation, virulence and response to stress were compared with the wild-type strain 70-15. The genes are named as *FZC1*∼*FZC87* (Fungal-specific Zn_2_ Cys_6_ transcription factor 1 to 87) if not designated in the succeeding text. The detailed phenotypic information of mutants was shown in Table S4 in Text S1.

We identified the homologs of the 104 *M. oryzae* Zn_2_Cys_6_ TF genes in *N. crassa* and *F. graminearum* by Blastp (www.broadinstitute.org), and then compared the phenotypes of the knockout mutants with the homologs studied in *N. crassa* [29] or *F. graminearum* [27] (Table S5 in Text S1). Deletion of members of the Zn_2_Cys_6_ TF family in *F. graminearum* and *N. crassa* resulted in mutant phenotypes at 16% (46/296) and 42% (30/72) of the mutants [27], [29]. However, more mutants (58.7%) in *M. oryzae* displayed visible mutant phenotypes. But only two mutants showed defects in growth and asexual and/or sexual development in three species simultaneously (Table S5 in Text S1). This comparison of mutant phenotypes between the three fungi suggested that the large majority of Zn_2_Cys_6_ TF genes evolved to have a unique, not conserved function in regulating fungal development.

### Zn_2_Cys_6_ transcription factors involved in fungal growth

**Figure 3:**
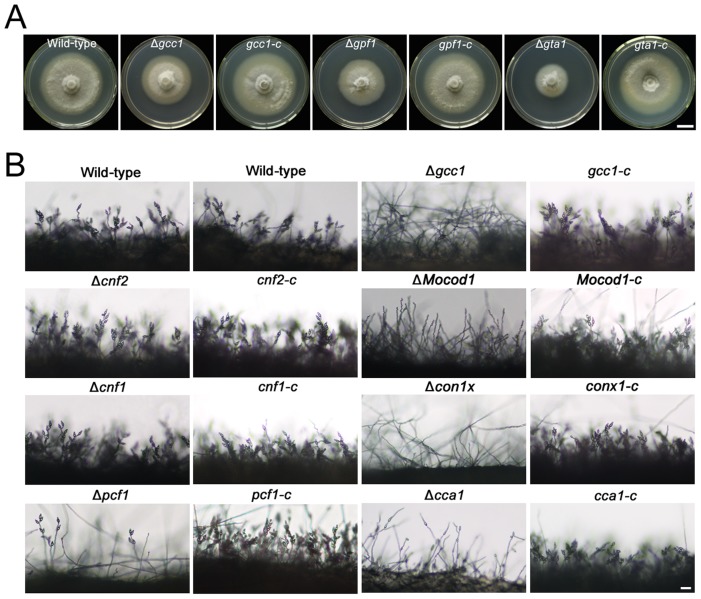
Colony growth and conidiophores of *M. oryzae* strains. (**A**) Colony growth of the wild-type strain 70-15, mutants (Δ*gcc1*, Δ*gpf1* and Δ*gta1*), and complemented strains (*gcc1*-c, *gta1*-c, *gta1*-c) on CM medium for 8 days. Bar = 1 cm. (**B**) Conidiophores of the wild-type, mutants (Δ*cnf1*, Δ*cnf2*, Δ*pcf1*, Δ*cca1*, Δ*conx1*, Δ*gcc1* and Δ*Mocod1*), and complemented strains (*cnf1-c*, *cnf2-c*, *pcf1-c*, *cca1-c*, *conx1-c*, *gcc1-c* and *Mocod1-c*). Bar = 20 µm.

The null mutants of 27 Zn_2_Cys_6_ TFs showed significant differences in fungal growth with the wild-type strain (Figure 2B, Table 1). Among these null mutants, three mutants (01C9-1/Δ*MGG_07063*, 01F1-1/Δ*MGG_17841* and 02G4-1/Δ*MGG_07149*) showed reduced colony growth on CM medium at 24.7, 26.6 and 43.5%, respectively (Table S4 in Text S1). The TF genes deleted in these mutants were designated *GCC1* (growth, conidiation and cell wall regulatory factor 1, MGG_07063), *GPF1* (growth and pathogenicity regulatory factor 1, MGG_17841) and *GTA1* (growth and tolerance to acidic stress regulatory factor 1, MGG_07149), respectively. To confirm the defects of the mutants in growth were caused by the knockout of the TF genes, we complemented the three mutants Δ*gcc1*, Δ*gpf1* and Δ*gta1* with their respective native copies from wild-type strain 70-15 (Figure S4C). The phenotypic analyses showed the *GCC1*-rescued strain (*gcc1*-c) and *GTA1*-rescued strain (*gta1*-c) all recovered from their defects in growth, and *GPF1*-rescued strain (*gpf1-c*) also recovered most defects in growth when compared to the mutants and the wild-type strain (Table 2, Figure 3A). These results implied that Zn_2_Cys_6_ TFs *GCC1*, *GPF1* and *GTA1* were required for the regulation of fungal growth in the rice blast fungus.

**Table 2:** Developmental characteristics of *M. oryzae* strains.

Strain	Growth (mm)	Conidiation (×10^3^/cm^2^)	Conidial germination (%)	Appressorium formation (%)	Strain	Growth (mm)	Conidiation (×10^3^/cm^2^)	Conidial germination (%)	Appressorium formation (%)
Wild-type	48.7±0.6A*	62.5±10.0C	95.2±1.8A	95.9±1.6A					
Δ*gcc1*	38.7±0.6C	0.0±0.0E			*gcc1-c*	48.7±0.6A	61.0±8.3C		
Δ*gpf1*	38.0±0.1C		76.4±6.4C	86.5±1.1C	*gpf1-c*	47.0±1.0B		94.0±1.5A	97.3±0.7A
Δ*gta1*	27.3±0.6D		84.5±0.5B	97.8±0.3A	*gta1-c*	49.7±0.6A		92.5±2.6A	97.8±1.6A
Δ*cnf1*		1229.7±101.1A	86.7±2.7B	89.9±3.5B	*cnf1-c*		66.4±8.8C	91.8±2.4A	96.2±0.3A
Δ*cnf2*		188.9±7.6B			*cnf2-c*		71.0±5.2C		
Δ*Monit4*		20.2±4.6D			*Monit4-c*		59.3±15.6C		
Δ*pcf1*		28.5±6.1D	86.4±2.4B	87.4±2.5B	*pcf1-c*		58.6±8.0C	94.4±0.9A	97.1±2.1A
Δ*cca1*		7.3±1.7E	3.6±0.6E	10.2±0.8E	*cca1-c*		65.9±6.4C	94.4±1.7A	96.8±0.7A
Δ*conx1*		0.1±0.0E			*conx1-c*		65.7±4.4C		
Δ*Mocod1*		0.0±0.0E			*Mocod1-c*		60.8±10.3C		
Δ*fzc16*			84.6±3.0B	97.1±1.2A	*fzc16-c*			95.1±1.8A	98.5±0.8A

Notes: The strains (5-mm mycelial blocks) were grown on CM medium for 8 days, and the diameter of colonies was then measured (growth) or conidia were collected and counted (conidiation). 40-µl (1×10^5^ conidia/ml) conidial suspensions were incubated on plastic slides for 4 hpi (conidial germination assay) and 24 hpi (appressorium formation assay).

*Same capital letters indicate non-significant difference estimated by Duncan's test in each developmental item (*P*≤0.05).

The experimental strains were the wild-type strain, the mutants (Δ*cca1*, Δ*conx1*, Δ*cnf1*, Δ*cnf2*, Δ*fzc16*, Δ*gcc1*, Δ*gpf1*, Δ*gta1*, Δ*Mocod1*, Δ*Monit4* and Δ*pcf1*) and complemented strains (*cca1-c*, *conx1-c*, *cnf1-c*, *cnf2-c*, *fzc16-c*, *gcc1*-c, *gpf1-c*, *gta1-c*, *Mocod1-c*, *Monit4-c* and *pcf1-c*).

### Zn_2_Cys_6_ transcription factors involved in fungal asexual development

Among the 104 Zn_2_Cys_6_ TFs, the null mutants of 25 genes displayed distinct changes in asexual development (Figure 2B, Table 1). Of these mutants, 21 mutants produced less conidia or even no conidia, but the other 4 mutants produced more conidia (Table 1, Table S4 in Text S1). We selected 2 mutants without conidiation, 4 mutants with less conidiation and 2 mutants with more conidiation to be complemented by their native genes to show the relationship between mutant's phenotype and TF gene deleted (Figure S4C). The genes deleted in these null mutants were *CNF1* (conidial production negative regulatory factor 1, MGG_02962 in mutant 06B5-7), *CNF2* (conidial production negative regulatory factor 2, MGG_15023 in mutant 03C7-2), *PCF1* (pathogenicity and conidiation regulatory factor 1, MGG_17623 in mutant 03C11-4), *MoNIT4* (a homolog of the *nit-4* gene, which is a pathway-specific regulatory gene that mediates nitrate induction in *N. crassa* [38], MGG_01518 in mutant 01A7-1), *CCA1* (conidiation, conidial germination and appressorium formation required transcription factor 1, MGG_05659 in mutant 06C2-14), *CONx1* (conidiation required transcription factor x1, MGG_12349 in mutant 06D5-21), *GCC1* (MGG_07063 in mutant 01C9-1) and *Mocod1* (homolog of amyR from *Aspergillus nidulans* [3], [39], [40], MGG_05343 in mutant 02F2-6). The mutants Δ*cnf1* and Δ*cnf2* produced more conidia than did the wild-type; mutants Δ*pcf1*, Δ*cca1* and Δ*conx1* produced less conidia; and mutants Δ*gcc1* and Δ*Mocod1* did not produce any conidia. The complementation experiments displayed that the defects of mutants in conidiation were caused by the deletion of the TF genes, since the complemented strains of these mutants all recovered from their abnormal asexual developmental phenomena (Table 2, Figure 3B).

**Figure 4:**
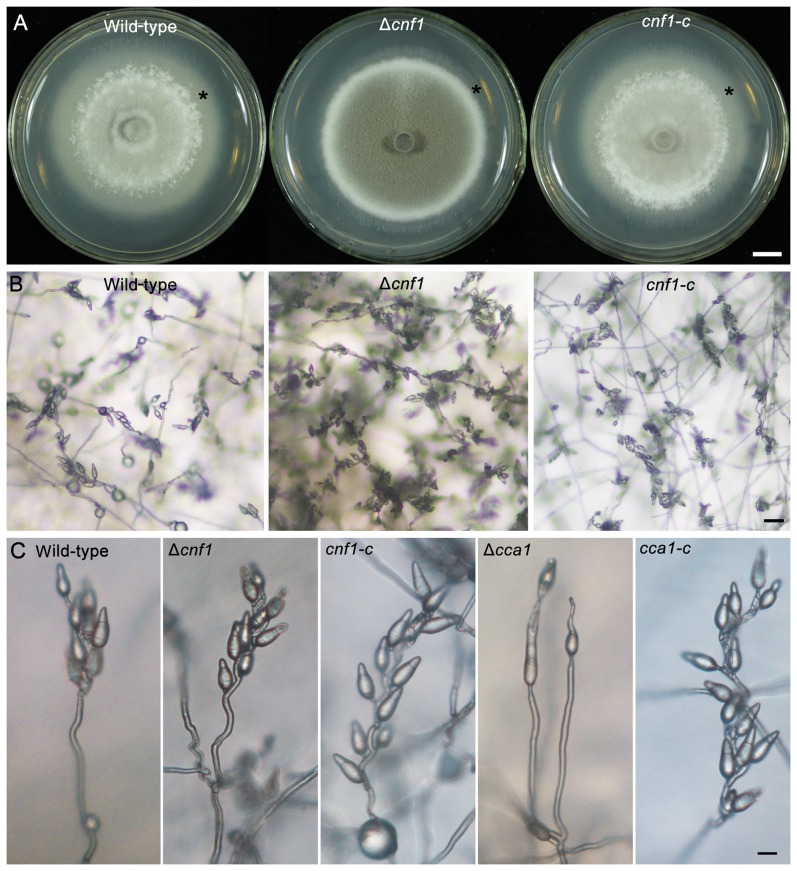
Mycelial appearance and spore-bearing aerial hyphae of the *M. oryzae* strains. (**A**) The mycelial appearance of the wild-type strain, Δ*cnf1* and its complemented strain *cnf1-c*. Stars indicate non-sporulating hyphae. Bar = 1 cm. (**B**) Spore-bearing aerial hyphae of the wild-type strain, Δ*cnf1* and its complemented strain *cnf1-c* on CM medium. Bar = 20 µm. (**C**) Conidial development on conidiophores of the *M. oryzae* strains Δ*cnf1* and Δ*cca1*. The conidiophore pictures of the wild-type, mutants (Δ*cnf1* and Δ*cca1*), and their complemented strains (*cnf1-c* and *cca1-c*) were taken on the strains grown on CM medium on microscope slides for 1 day. Bar = 40 µm.

*CNF1* is a strong conidial production negative regulatory factor in rice blast fungus, and its deletion may result in greatly increased conidial production (usually 20- to 40-fold of the wild-type strain). Moreover, the mutant Δ*cnf1* had significant changes in mycelial appearance, such as deep dark colony color from the gray-white of the wild-type strain, short spore-bearing aerial hyphae from long aerial hyphae of the wild-type strain, and spore-bearing aerial hyphae formed at the rim of mycelial colony earlier than in the wild-type strain (Figure 4A). Although the conidiophore development of Δ*cnf1* seemed similar to that of the wild-type strain and *CNF1*-rescued mutant *cnf1-c* (Figure 3B, Figure 4C), more aerial hyphae differentiated into conidiaophores in Δ*cnf1* (Figure 4B). The increase in conidial production of Δ*cnf1* was possibly partly due to the spore-bearing hyphae differentiating earlier and more from the aerial hyphae than in the wild-type strain.

**Figure 5:**
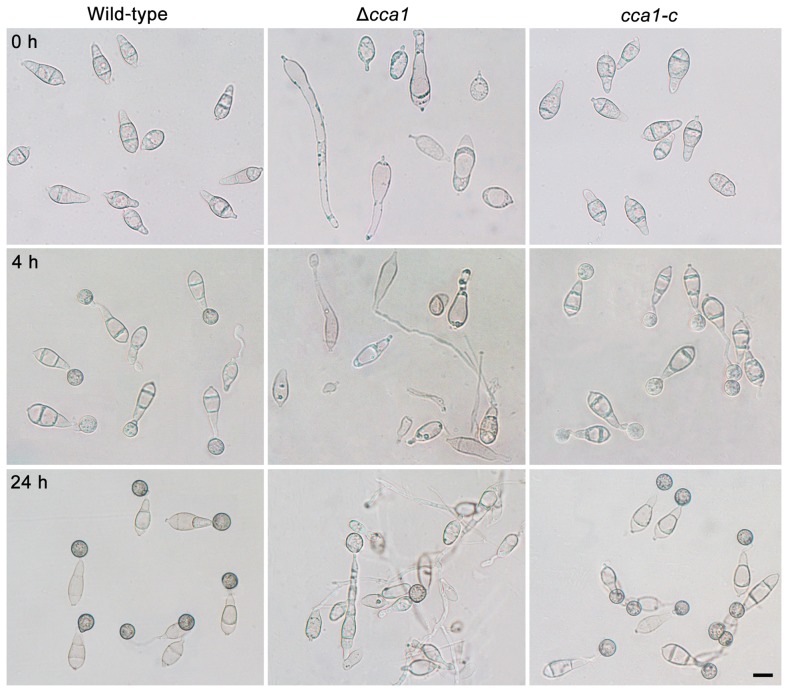
Conidia, conidial germination and appressorium formation of the *M. oryzae* strain Δ*cca1*. The conidia (0 h) of the wild-type strain, mutant Δ*cca1*, and its complemented strain *cca1-c* were grown in H_2_O on plastic cover slides for 4 h (conidial germination) and 24 h (appressorium formation). Bar = 10 µm.

Δ*cca1* produced few and odd, long and vacuolated conidia (Table 2, Figure 5). Single or two tandem spores differentiated from the apex of conidiophores in the mutant Δ*cca1*, and the *CCA1*-rescued mutant *cca1-c* produced normal conidiophores and conidia similar as the wild-type strain (Figure 4C, Figure 5). These results suggested that *CCA1* is required for the differentiation of conidiophores and the formation of conidia.

### Zn_2_Cys_6_ transcription factors involved in conidial germination and appressorium formation

When the null mutants of 101 Zn_2_Cys_6_ TFs were assayed for their conidial germination rate at 4 hpi (hour post inoculation) and their appressorium formation rate at 24 hpi, 12 mutants and 10 mutants displayed significant differences in conidial germination and appressorium formation, respectively (Figure 2B, Table 1). We assayed conidial germination and appressorium formation in the complemented strains of 6 null mutants (Δ*fzc16*, Δ*gpf1*, Δ*gta1*, Δ*pcf1*, Δ*cnf1* and Δ*cca1*) (Figure S4C) and found that these complemented strains all recovered from their defects in conidial germination or in appressorium formation (Table 2). These experiments implied that Zn_2_Cys_6_ TFs *GPF1*, *PCF1*, *CNF1*, and *CCA1* are required for conidial germination and appressorium formation and that *FZC16* and *GTA1* are required for conidial germination in the rice blast fungus.

Among these 6 mutants, the mutant Δ*cca1* showed marked defects not only in conidial germination and appressorium formation, but also in conidiation (Table 2). The conidial germination rate of the mutant Δ*cca1* was 3.6%, and appressorium formation rate was 10.2% (Table 2). The structure and shape of conidium, germinated conidium and appressorium of the mutant Δ*cca1* displayed notable differences with the wild-type strain (Figure 5). Therefore, the mutant Δ*cca1* produced few multifarious and vacuolated conidia, which germinated and formed not fully melaninized appressoria at a low ratio.

### Zn_2_Cys_6_ transcription factors required for virulence

The pathogenicity of 104 null mutants was tested first on intact barley leaves by placing agar plugs containing mycelia on them. Mutant Δ*gpf1* (MGG_17841) was found to lose pathogenicity to barley, and 4 mutants Δ*cnf1* (MGG_02962), Δ*cnf2* (MGG_15023), Δ*Mocod1* (MGG_05343) and Δ*pcf1* (MGG_17623) showed reduced pathogenicity (Table S4 in Text S1, Figure S2). We then determined the pathogenicity of 101 mutants on rice seedlings (CO-39) by spraying with conidial suspension (1×10^5^ spores/ml). The results showed that it was decreased in 3 mutants Δ*cnf1* (MGG_02962), Δ*cnf2* (MGG_15023) and Δ*gta1* (MGG_07149) and that 2 mutants Δ*gpf1* (MGG_17841) and Δ*cca1* (MGG_05659) lost their virulence to rice (Table S4 in Text S1, Figure S3A). Impressively, Δ*cca1* caused lesions on barley leaves when inoculated with mycelial plugs, but lost virulence on rice seedlings when spayed as a conidial suspension. The other 3 mutants Δ*conx1* (MGG_12349), Δ*gcc1* (MGG_07063) and Δ*Mocod1* (MGG_05343) with little or no conidiation were tested on intact rice leaves with agar plugs containing mycelia. Δ*conx1* and Δ*Mocod1* were found to have lower virulence (Table S4 in Text S1, Figure S3B).

**Figure 6:**
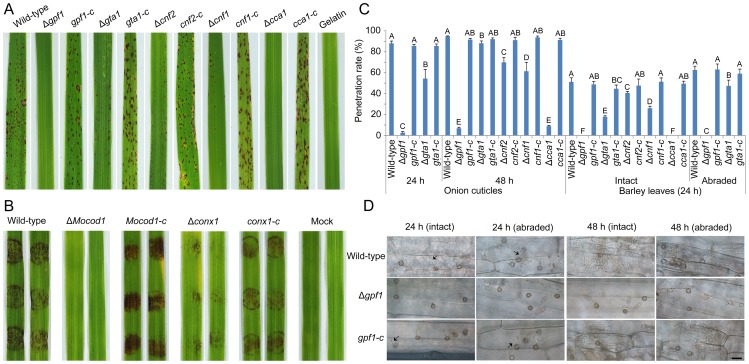
Pathogenicity assay of the *M. oryzae* strains. (**A**) Pathogenicity assay of 5 mutants and their complementation strains on rice. These 5 mutants are Δ*gpf1*, Δ*gta1*, Δ*cnf2*, Δ*cnf1*, Δ*cca1* and their complementation strains *gpf1-c*, *gta1-c*, *cnf2-c*, *cnf1-c*, and *cca1-c*. The rice seedlings were sprayed with a conidial suspension of *M. oryzae* strains and cultured for 7 days. (**B**) Pathogenicity assay of the mutants on rice leaf explants. The mycelial agar plugs of the mutants Δ*Mocod1* and Δ*conx1*, their complementation strains *Mocod1-c* and *conx1-c*, and the wild-type strain 70-15 were placed on intact rice leaves for 4 days. (**C**) Penetration assay. 10-or 20-µl (5×10^4^ conidia/ml) conidial suspensions were inoculated on onion cuticle or barley leaves (intact or abraded) and incubated for 24 or 48 h. The experimental strains were the wild-type strain 70-15, mutants (Δ*gpf1*, Δ*gta1*, Δ*cnf2*, Δ*cnf1* and Δ*cca1*) and complemented strains (*gpf1-c*, *gta1-c*, *cnf2-c*, *cnf1-c* and *cca1-c*). Same capital letters in same treatment item indicate non-significant difference estimated by Duncan's test (*P*≤0.05). (**D**) Penetration of Δ*gpf1*. 20 µl (5×10^4^ conidia/ml) conidial suspensions of Δ*gpf1*, complemented strain *gpf1-c* or wild-type strain were inoculated on barley leaf explants (intact or abraded) and incubated for 24 or 48 h. Arrows indicate the *in planta* hyphae invaded. Bar = 25 µm.

After complemented with native genes (Figure S4C), we retested the pathogenicity of the 7 mutants with their complemented strains on rice seedlings by spraying with conidial suspensions (Δ*gpf1*, Δ*cnf1*, Δ*cnf2*, Δ*gta1*, Δ*cca1* and *gpf1-c*, *cnf1-c*, *cnf2-c*, *gta1-c*, *and cca1-c*) (Figure 6A) or on rice leaf explants by inoculating with mycelial plugs (Δ*Mocod1*, Δ*conx1* and *Mocod1-c*, *conx1-c*) (Figure 6B). The facts that the complemented strains recovered from their defects in virulence on rice implied that the loss or weakening of virulence in the mutants was caused by the deletion of the TF genes *GPF1*, *CNF1*, *CNF2*, *GTA1*, *CCA1*, *CONx1* and *MoCOD1*, respectively. To assay the roles of these TF genes in plant penetration, onion cuticle and barley leaves were inoculated with a conidial suspension of the mutants and their complemented strains. Compared with the wild-type strain, the ability of Δ*gpf1*, Δ*gta1*, Δ*cnf2*, Δ*cnf1* and Δ*cca1* to penetrate the onion cuticle and barley leaf cuticle was weakened to some extent (Figure 6C). Notably, the mutants Δ*gpf1* and Δ*cca1* were unable to penetrate barley cuticle, even after barley cuticle was abraded (for Δ*gpf1*), while the wild-type strain penetrated into the epidermal cells of barley leaves and grew invasively after 24 hpi (Figure 6C, Figure 6D). These results suggested that *GPF1* is required by the rice blast fungus for penetration into the plant cuticle and possibly for invasive growth. Although the conidia of Δ*cca1* were unable to penetrate barley cuticle and could not cause lesions in rice, the mycelia of Δ*cca1* could cause lesions in barley and rice. The difference in virulence between conidia and mycelia may lie in the conidial defects of Δ*cca1* in germination, appressorium formation and host penetration ability.

### Phenotypic analyses of Zn_2_Cys_6_ transcription factor deletion mutants under stress conditions

There are various stress conditions encountered by *M. oryzae* when it completes its infection cycle in rice. It is possible to predict the biological functions of these deleted Zn_2_Cys_6_ TF genes in cell activity regulation by assaying the resistance ability to stress conditions of the mutants. We screened and assayed in a rapid way the mycelial growth of the mutants under 9 abiotic stress conditions (0.5 mM H_2_O_2_, 0.005% SDS, 0.3 M CaCl_2_, 0.5 M NaCl, 1 M sorbitol, olive oil as a single organic carbon source, pH 5.0 and pH 9.0, and minimal medium). Four mycelial inoculation blocks were placed in a 9-cm plate by needle point inoculation and the diameter of mycelial colonies were measured after 6 days culture. The results displayed that many mutants had significant visible phenotypes under those stress conditions when compared with the wild-type strain (Table S4 in Text S1): 14 mutants showed mutant phenotypes in MM medium (nitrogen metabolism), 16 mutants in CM-C medium containing olive oil (carbon metabolism), 22 mutants in CM medium with SDS (cell wall stress), 17 mutants or 19 mutants in CM medium with high concentration of Ca^2+^ or Na^+^ (ionic regulation), 18 mutants in CM medium with 1 M sorbitol (hypertonic pressure), 30 mutants in CM medium with 0.5 mM H_2_O_2_ (oxidative stress), and 30 mutants or 14 mutants in CM medium at pH 5.0 or pH 9.0 (ambient pH regulation).

**Figure 7:**
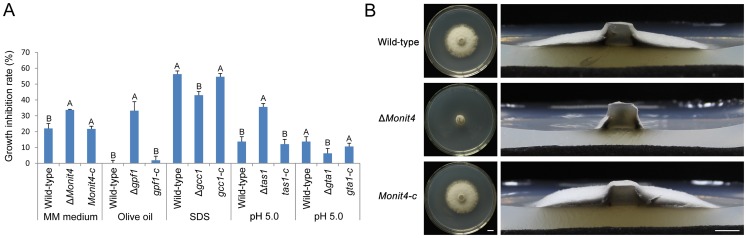
Growth of the *M. oryzae* strains under stress conditions. (**A**) Growth inhibition rate. 5-mm mycelial blocks of *M. oryzae* strains were inoculated on CM medium and medium with stress conditions for 8 days, and the diameter of colonies was then measured to calculate the growth inhibition rate. The experimental strains were the wild-type strain, mutants (Δ*Monit4*, Δ*gpf1*, Δ*gcc1*, Δ*tas1* and Δ*gta1*), and complemented strains (*Monit4-c*, *gpf1-c*, *gcc1-c*, *tas1-c* and *gta1-c*). Same capital letters in same stress item indicate non-significant difference estimated by Duncan's test (*P*≤0.05). (**B**) Mycelial growth of Δ*Monit4* on MM medium. The wild-type strain, Δ*Monit4*, and complemented strain *Monit4-c* were grown on MM medium at 25°C for 8 days. Bar = 5 mm.

After comparison of the relative growth rate of mutants on CM medium, we selected 5 mutants (01A7-1, 01B11-1, 01C9-1, 01F1-1 and 02G4-1) that had significant changes in mycelial growth under stress conditions to be complemented with native genes to see if these phenotypes were caused by the deletion of TF genes (Figure S4). The TF genes in these 5 mutants were: *MoNIT4* in mutant 01A7-1 (MGG_01518), *GPF1* in mutant 01F1-1 (MGG_17841), *GCC1* in mutant 01C9-1 (MGG_07063), *TAS1* (tolerance to acidic stress regulatory factor 1) in mutant 01B11-1 (MGG_04108) and *GTA1* in mutant 02G4-1 (MGG_07149). The mycelial growth of Δ*Monit4* in minimal medium, Δ*gpf1* in CM-C medium containing olive oil, and Δ*tas1* at pH 5.0 was reduced (Figure 7A). However, the mycelial growth of Δ*gcc1* in CM medium containing SDS and Δ*gta1* at pH 5.0 was increased. When rescued with native genes, these mutants all recovered their growth under stress conditions (Figure 7A). Interestingly, the mutant Δ*Monit4* grew normally as the wild-type strain on CM medium (Table S4 in Text S1, Figure S1), but grew slowly as a very sparse and thin mycelium layer on MM medium (Figure 7B). As previously reported, *MoNIT4* is necessary for the regulation of nitrogen metabolism [38], [41]. These data also suggested that TF gene *GPF1* is necessary for gene expression regulation relative to fat metabolism, that *GCC1* is necessary for gene expression regulation relative to cell wall synthesis, and that *TAS1 and GTA1* are necessary for gene expression regulation in response to acidic stress.

### Genes regulated by two virulence-required genes *GPF1* and *CNF2*

Eight Zn_2_Cys_6_ TF genes (*CCA1*, *CNF1*, *CNF2*, *CONx1, GPF1*, *GTA1*, *MoCOD1* and *PCF1*) are required for fungal pathogenicity to rice or barley. Δ*gpf1* and *Δcnf2* are two mutants that have similar phenotypes on virulence with relatively limited differences in other fields, because Δ*Mocod1* and Δ*conx1* do not produce conidia, Δ*cca1* and Δ*gta1* have virulence when inoculated on rice leaves with mycelial plugs, Δ*pcf1* shows reduced virulence on barley inoculated with mycelial plugs, but normal when sprayed on rice, and Δ*cnf1* produces greatly increased conidia. To understand how these TF genes affect fungal pathogenicity, the two genes *GPF1* and *CNF2*, whose mutants lose (Δ*gpf1*) or have reduced (Δ*cnf2*) virulence to both barley and rice, were selected to analyze their functions in gene expression regulation. Since Δ*gpf1* could not penetrate rice cuticle, we could not obtain its mRNA during infection. The rice blast fungus experiences temporal nitrogen starvation when it infects the plant, and the gene expression pattern of the mycelia under starvation is similar to that at plant infection [42]–[45], and the hyphae enduring starvation are similar to those inoculated as mycelial plugs in plant infection. We therefore analyzed the genome-wide gene expression of the mycelia after 4 h starvation of the wild-type strain, Δ*gpf1* and Δ*cnf2* by RNA-seq. The RNA-seq experiments were performed in biologic triplicate for each strain.

**Figure 8:**
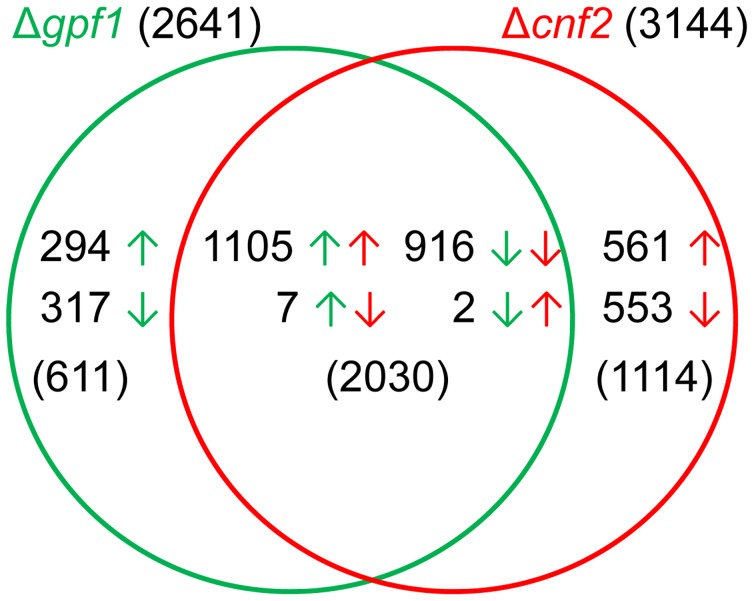
Comparison of differentially expressed genes between Δ*gpf1* and Δ*cnf2*. Green or red arrows are the directions of gene expression regulation in Δ*gpf1* or Δ*cnf2*.

The transcripts of 10,864, 11,048 and 10,681 genes were identified in the starved mycelia of the wild-type strain, Δ*gpf1* and Δ*cnf2*, respectively. Compared with the wild-type strain, 2641 genes were differentially expressed significantly in Δ*gpf1* (FDR<0.05), with 1406 genes up-regulated and 1235 down-regulated (Table S6 in Text S1), and 3144 genes in Δ*cnf2* (FDR<0.05), with 1668 genes up-regulated and 1476 down-regulated (Table S7 in Text S1). The similarities and differences between genes regulated by *GPF1* and *CNF2* were analyzed in detail by comparing the DEGs in Δ*gpf1* or Δ*cnf2*. The comparison assays showed that the expression of 611 genes or 1114 genes were independently regulated by *GPF1* or *CNF2*, while 2030 genes were regulated by both *GPF1* and *CNF2* together (Figure 8). Among the 2030 genes commonly regulated by *GPF1* and *CNF2*, 916 genes were down-regulated and 1105 genes up-regulated simultaneously in both Δ*gpf1* and Δ*cnf2*, while only 9 genes were regulated in the opposite direction in both mutants (Figure 8, Table S8 in Text S1). It is very surprising that the expression of so many DEGs were regulated in the same direction in Δ*gpf1* and Δ*cnf2* (Figure 8). The correlation coefficient between DEGs of Δ*gpf1* and Δ*cnf2* was determined by the linear trend model, and the result showed R^2^ = 0.88 with *p*<0.0001. These similar patterns were not mainly caused by the starvation, as we also assayed the mutants of five TF genes containing non-Zn_2_Cys_6_ domains by RNA-seq with the same treatment at the same time, but the mutants displayed specific DEG patterns different from Δ*gpf1* and Δ*cnf2*. Therefore, these similar patterns in gene expression regulation in two mutants were possibly caused by the fact that TFs Gpf1 and Cnf2 had the same DNA-binding domain (Zn_2_Cys_6_ domain), and Δ*gpf1* or Δ*cnf2* had similar phenotypes in pathogenicity. However, *GPF1* and *CNF2* still showed great differences in gene regulation since 1152 DEGs appeared in Δ*cnf2* when compared with Δ*gpf1* (Table S9 in Text S1).

To see how *GPF1* and *CNF2* affect the pathogenicity to plants, we reviewed the functions of the DEGs which were studied in previous reports. Fifty DEGs in Δ*gpf1* and 68 DEGs in Δ*cnf2* (total of 80 DEGs in both were studied by knockout. Of the 80 genes, the mutants of 57 genes (36 DEGs in Δ*gpf1* and 49 DEGs in Δ*cnf2*) displayed defects in virulence (Table 3) but the mutants of 23 genes (14 DEGs in Δ*gpf1* and 19 DEGs in Δ*cnf2*) were dispensable for fungal pathogenicity (Table S10 in Text S1). Interestingly, of these reported pathogenicity-required genes, the number of down-regulated genes (45 DEGs) was much higher than that of up-regulated genes (12 DEGs) in the two mutants Δ*gpf1* and Δ*cnf2* (Table 3). These pathogenicity-required genes are mainly related to transcription (such as *CON7*, *MoLDB1*, *MoMIG1*, *MoPAC2*, *MSTU1*, *MST12* and *PTH12*) [8], [12], [18], [20], [21], [46], [47], G-protein signaling (such as *MAGB*, *MoRGS1* and *MGB1*) [48]–[51], MAPK pathway (such as *MST7*, *MoMCK1* and *MoMPS1*) [52]–[54], autophagy (such as *MoATG1*, *MoATG5*, *MoATG9* and *SNX41*) [55]–[58], regulation of reactive oxygen species (such as *MoHYR1* and *GTR1*) [59], [60], and amino acid, lipid and carbohydrate metabolism (such as *ILV2*, *MoSNF1*, *ICL1* and *MoPLAA*) [61]–[64]. We also checked the relationships between Zn_2_Cys_6_ TFs and Gpf1 or Cnf2 in transcription regulation. Surprisingly, 60 or 66 Zn_2_Cys_6_ TF genes were listed as DEGS of Δ*gpf1* or Δ*cnf2* (total of 79 genes in two mutants) (Table S11 in Text S1). The Zn_2_Cys_6_ TF genes are also the important targeted genes regulated by two Zn_2_Cys_6_ TF genes *GPF1* and *CNF2*. These facts implied that the well-balanced and sophisticated expression regulation of TF genes and pathogenicity-required genes is necessary for the maintenance of pathogenicity in *M. oryzae*.

**Table 3:** *GPF1*- or *CNF2*-dependent genes encoding known virulence factors.

Gene locus (MG8)	Gene	Δ*gpf1* a	Δ*cnf2* a	Virulence on plants/Function	Reference
MGG_00365	*MAGB*	-	−1.58	Reduced virulence/G-protein signaling	[48]
MGG_00466	*MoCDC42*	−1.52	−2.00	Reduced virulence/CDC42	[74]
MGG_00501	*TGD2*	−1.67	−2.68	Reduced virulence/Transcription factor	[15], [72], [83]
MGG_00692	*MSTU1*	−7.80	−11.85	Reduced virulence/Transcription factor	[8]
MGG_00800	*MST7*	−2.00	−1.67	No virulence/MAPK pathway	[52]
MGG_00803	*MoSNF1*	−1.43	−1.79	Reduced virulence/Glucose derepression pathway	[62]
MGG_00883	*MoMCK1*	−1.39	−1.52	No virulence/MAPK pathway	[53]
MGG_01057	*MoLDB1*	−1.63	−2.18	Reduced virulence/Transcription factor	[18]
MGG_01204	*MoMIG1*	-	−1.95	No virulence/Transcription factor	[20]
MGG_01230	*MoSSADH*	−1.54	-	Reduced virulence/γ-aminobutyric acid degradation	[23]
MGG_01282		1.46	2.26	No virulence/polyubiquitin	[84]
MGG_01558	*SET3*	-	−1.53	No virulence/Set3 complex	[85]
MGG_01662	*MoAAT*	-	−1.56	Reduced virulence/4-aminobutyrate aminotransferase	[23]
MGG_01663	*HOS2*	−1.46	−1.51	No virulence/Set3 complex	[85]
MGG_02531	*MoVPR*	−3.04	−3.00	Reduced virulence	[23]
MGG_02731	*MoRAC1*	-	−1.45	Reduced virulence/Small GTPase	[86]
MGG_02848	*MC69*	−1.57	-	Reduced virulence/Secreted proteins	[87]
MGG_02897	*Mossk1*	-	−1.81	Reduced virulence/Response regulator	[79]
MGG_03065	*MoVMA11*	−1.53	-	No virulence/ATPase	[46]
MGG_03694	*MoATG6*	−1.39	-	No virulence/Autophagy	[88]
MGG_04050	*MoSEC22*	−2.81	−3.80	No virulence/R-Snare	[89]
MGG_04895	*ICL1*	−1.72	−1.59	Reduced virulence/Glyoxylate cycle	[63]
MGG_04943	*MoMPS1*	-	−1.75	No virulence/MAPK pathway	[54]
MGG_05133	*MoCRZ1*	-	−1.42	Reduced virulence/Ca^2+^ signaling	[9], [90]
MGG_05201	*MGB1*	−1.67	−1.80	No virulence/G-protein signaling	[51]
MGG_05287	*CON7*	-	−1.93	No virulence/Transcription factor	[12]
MGG_05428	*MoVAM7*	-	−1.89	No virulence/Snare	[91]
MGG_05664	*MoPDEH*	-	−1.77	Reduced virulence/cAMP signaling	[92]
MGG_05755	*MoMON1*	1.38	-	No virulence/Vacuole and vesicle fusion	[93]
MGG_06033	*MoMSB2*	-	−1.78	Reduced virulence/Signaling	[94]
MGG_06249	*MoTCTP*	-	1.43	Reduced virulence/Mitochondrial protein	[80]
MGG_06393	*MoATG1*	−1.36	−1.51	No virulence/Autophagy	[55]
MGG_06439	*MoTEA4*	−1.88	−2.26	Reduced virulence	[95]
MGG_06564	*MoPAC2*	-	−1.67	Reduced virulence/Transcription factor	[96]
MGG_06868	*ILV2*	4.24	4.03	Reduced virulence/Amino acid metabolic pathway	[61]
MGG_06971	*MoSFL1*	-	−1.73	Reduced virulence/Transcription factor	[22]
MGG_07460	*MoHYR1*	1.73	2.32	Reduced virulence/Reactive oxygen species	[59]
MGG_08212	*MoATF1*	1.42	-	Reduced virulence/Transcription factor	[24]
MGG_08850	*MoGTI1*	−1.58	−2.55	Reduced virulence/Stress	[96]
MGG_08895	*GTR1*	2.06	1.82	Reduced virulence/Reactive oxygen species	[60]
MGG_09125	*MoSHO1*	-	−1.80	Reduced virulence/Signaling	[94]
MGG_09262	*MoATG5*	7.64	8.88	No virulence/Autophagy	[56]
MGG_09559	*MoATG9*	−1.41	−1.42	No virulence/Autophagy	[57]
MGG_09565	*PMK1*	−1.49	−1.93	No virulence/MAPK pathway	[52]
MGG_09912	*MoCMK1*	−1.75	−1.46	Reduced virulence/Ca^2+^ signaling	[97]
MGG_10315	*MPG1*	−12.49	−15.83	Reduced virulence/Hydrophobin	[75]
MGG_10323	*MoRHO3*	−1.46	−1.69	No virulence/Small GTPase	[98]
MGG_11346	*MoCDTF1*	-	−1.72	No virulence/Transcription factor	[19]
MGG_12828	*MoATG15*	1.48	-	No virulence/Autophagy	[88]
MGG_12832	*SNX41*	−1.36	-	No virulence/Autophagy	[58]
MGG_12865	*PTH12*	2.97	2.09	No virulence/Transcription factor	[16], [47]
MGG_12868	*ECH1*	-	1.47	Reduced virulence/Enoyl-CoA hydratase	[99]
MGG_12958	*MST12*	-	−1.71	No virulence/Transcription factor	[21]
MGG_13624	*MoABC1*	-	−1.59	No virulence/ABC transporter	[100]
MGG_14014	*MoPLAA*	2.12	2.09	Reduced virulence/Lipid metabolism	[64]
MGG_14517	*MoFLBA*	-	−1.69	Reduced virulence/G-protein signaling	[49], [50], [101]
MGG_14847	*MST11*	-	−1.33	No virulence/MAPK pathway	[52]

Note:

a ) The expression of genes increased or decreased in Δ*gpf1* and Δ*cnf2*.

The number means the fold change, and the symbol “−” means no significant change (FDR<0.05) afterwards compared with the wild-type strain.

## Discussion

Since the genomes of a large number of fungi have been sequenced and are being sequenced, there is a surge of interest in functional genomics research through the systematic mutagenesis of identified genes. The construction of a genome-wide gene deletion mutant set of fungi based on the homologous recombinational gene knockout procedure is a valuable resource for the analysis of fungal development, pathogenicity, and many aspects of cell biology and biochemistry, such as those done in the budding yeast *S. cerevisiae* [65], the fission yeast *Schizosaccharomyces pombe* [66], and the saprobe filamentous fungus *N. crassa* [29]. However, except for *N. crassa*, *F. graminearum* and Aspergilli which are highly efficient in gene deletion experiments, no efficient system has been available to perform high-throughput gene knockout in filamentous fungi. In this study, we present an approach to knockout large numbers of genes that utilizes several methods suitable to high-throughput manipulation. Several time-consuming steps of the gene knockout procedure could be performed in a high-throughput way. In this procedure, the gene-deletion cassettes were built using a yeast homologous recombination method [29] with a yeast-*Escherichia-Agrobacterium* shuttle vector pKO1B, which is the first reported artificial plasmid that could be replicated in yeast, *E. coli* and *Agrobacterium* cells. The gene-deletion cassettes obtained in a binary vector pKO1B could be directly used to transform fungal cells through the ATMT method [67]. This advantage avoids the tedious and inefficient work to transfer the gene-deletion cassettes from a yeast plasmid to another binary *Agrobacterium* plasmid. The use of GFP fluorescence as a negative marker to eliminate most ectopic insertion transformants reduced the number of transformants one has to identify by PCR or Southern blot. The employment of the negative (for the targeted gene)/positive (for the unique recombinational DNA) identification PCR and qPCR to identify null mutants instead of Southern blot, which is not high throughput, makes the identification of null mutants a high-throughput procedure. The copies of the gene-deletion cassette in mutant genomic DNA quantified by qPCR has been widely performed in animals and plants [68], [69]. The reliability and stability of PCR or qPCR were guaranteed by the extraction of high quality genomic DNA with CTAB [70] performed in a high-throughput way in this study. The result of the gene knockout events (Figure S4A) were also reconfirmed by Southern blot at the DNA level in 10 mutants (Figure S4B) and at the transcript level in 16 mutants (Figure S4C). As a result, two researchers were able to complete one cycle of the gene knockout experiment for 96 genes in a month, including preparing knockout vectors, performing ATMT transformation, and identifying null mutants, which is a major improvement over the individual gene knockout protocol. More importantly, this procedure can be adapted for knocking out genes in other fungi without any modification or after the substitution of organism-specific promoter to drive the GFP reporter. Besides the *GFP* gene, a second drug resistance gene (*HPH*/hygromycin B phosphotransferase gene, *NEO*/neomycin phosphotransferase II gene or *BAR*/glufosinate resistance gene, etc.) or a herpes simplex virus thymidine kinase (HSVtk) gene, which converts 5-fluoro-2′-deoxyuridine to a toxic compound [71], could be used as an alternative negative selection marker against ectopic transformants. As an alternative vector, pKO1B-HPH (shown in Materials and Methods) is another yeast-*Escherichia-Agrobacterium* shuttle which could be used in this high-throughput gene knockout system besides pKO1B. To increase knockout efficiency, *M. oryzae KU80* null mutants, which are defective for non-homologous end joining (NHEJ) DNA repair [31], could be alternative strains used in targeted gene replacement instead of the wild-type strain.

In this study, we generated 104 fungal-specific Zn_2_Cys_6_ TF gene-deleted mutants in *M. oryzae* by a high-throughput gene knockout procedure and analyzed the phenotypes of individual TF mutants. The deletion of Zn_2_Cys_6_ TF genes resulted in phenotype changes in fungal development and pathogenicity in 58.7% mutants compared to the wild-type strain, while 26 mutants were defective in pleiotropic phenotype. Colony growth and asexual reproduction (conidiation) were the two phenotypic categories most observed in mutants, and most mutants defective in conidiation were often defective in vegetative growth (conidial germination, colony growth, pigmentation and mycelial appearance). Seven Zn_2_Cys_6_ TF genes functional for pathogenicity in *M. oryzae* are also especially required for vegetative growth, conidiation or appressorium formation. Comparison of phenotypes between *M. oryzae*, *F. graminearum* and *N. crassa* Zn_2_Cys_6_ TF gene orthologs seemed to agree with the previously reported viewpoint that Zn_2_Cys_6_ TFs evolved divergently in how to regulate fungal growth and asexual development, rather than keeping the same function in different fungi [27]. These divergences in functions may be due to the differences in the life cycles between the three fungi. *N. crassa* is an obligate saprophyte which lives on dead organic material and cannot attack a living host, while *M. oryzae* and *F. graminearum* are facultative saprophytic plant pathogens that additionally need specialized structures (such as an appressorium) and functions to infect and obtain nutrients from living plants.

Until now, 6 Zn_2_Cys_6_ TF genes (*MoCOD1*, *MoCOD2*, *MoNIT4*, *PIG1, TRA1* and *XLR1*) have been identified in the rice blast fungus [17], [39], [41], [72], [73], and 4 of them were also in our Zn_2_Cys_6_ TF gene knockout mutant set. The expression of *MoNIT4* and *MoCOD1* were up-regulated during conidiation [41], and their mutants Δ*Monit4* and Δ*Mocod1* had reduced conidiation [39], [41], and Δ*Mocod1* also had lower pathogenicity [39]. In our study, the mutants Δ*Monit4* and Δ*Mocod1* were also defective in conidiation or pathogenicity; furthermore, our mutant Δ*Mocod1* did not produce any conidia on CM medium. Similar to a previous report [74], our Δ*Tra1* mutant also showed defects in conidial germination. *PIG1* was identified to be involved in melanin biosynthesis, but not confirmed by knockout [73]; however, the Δ*pig1* mutant did not show notable phenotypes in fungal development in our study. These small discrepancies in mutant phenotypes with previously reported data may be due to the differences in wild-type strains and experimental conditions.

TFs Mnh6, Moatf1, Mocrz1 and Mstu1 were reported to regulate hyphal growth in the rice blast fungus, and the colony growth of the mutants Δ*mnh6*, Δ*Moatf1*, Δ*Mocrz1* and Δ*Mstu1* was about 70–90% of the wild-type strain [7]–[9], [24]. We found 27 Zn_2_Cys_6_ TF genes involved in fungal growth, and 3 genes *GCC1*, *GPF1* and *GTA*1 were required for normal colony growth. Interestingly, the colony growth of Δ*gta1* was only about 56% of that of wild-type strain, and *GTA1* is a gene which affects colony growth the most among known TF genes in the rice blast fungus. *GTA1* also functions in conidiation and pathogenicity to plants.

The change in asexual reproduction in a mutant is a phenotype that mostly happens when TF genes or other genes are deleted in the rice blast fungus. The TF gene-deleted mutants (Δ*com1*, Δ*con7*, Δ*cos1*, Δ*mnh6*, Δ*Mohox2*) showed reduced conidiation [7], [11]–[13], [15], [16]. In our study, 25 Zn_2_Cys_6_ TF genes were identified as being involved in fungal conidiation. Of them, *CCA1*, *GCC1*, *MoCOD1* and *CONx1* are necessary for the differentiation of conidiophores. Unexpectedly, the deletion of four Zn_2_Cys_6_ TF genes (*CNF1, CNF2, CNF3 and CNF4*) led to increased conidial production. In particular, Cnf1 is the strongest negative regulatory factor of conidial production identified in the rice blast fungus until now. The deletion of *CNF1* led to earlier and more differentiation of spore-bearing hyphae than in the wild-type strain. Impressively, the mutants Δ*cnf1* and Δ*cnf2* produced more conidia, but their conidia had reduced virulence to barley and rice. Conidia are the main way to spread the rice blast disease. It is clear that a balance exists between the ability to produce conidia and the pathogenicity to plant in the phytopathogenic fungus.

We identified 2641 genes regulated by *GPF1* and 3144 genes by *CNF2* through RNA-seq. Interestingly, 2021 genes were regulated in the same direction in Δ*gpf1* and Δ*cnf2*. This fact suggested *GPF1* and *CNF2* have similar mechanisms in the regulation of fungal pathogenicity. Nearly sixty DEGs in Δ*gpf1* and Δ*cnf2* were confirmed to be required for fungal pathogenicity (Table 3). In particular, the expression of two pathogenicity-required genes, an APSES TF gene *MSTU1* [8] and a hydrophobin gene *MPG1* [75], were greatly down-regulated in Δ*gpf1* (7.80- and 12.49- fold, respectively) and Δ*cnf2* (11.85- and 15.83-fold, respectively). About half of Zn_2_Cys_6_ TF genes and many other DNA-binding domain TF genes were regulated by *GPF1* and *CNF2*. These data primarily revealed the gene expression network in the regulation of pathogenicity controlled by *GPF1* and *CNF2*. It is necessary to keep the well-balanced expression of TF genes and pathogenicity-required genes for maintenance of pathogenicity in *M. oryzae*.

The high-throughput analysis of mutant phenotypes showed that some TF gene mutants shared highly overlapping phenotypes, such as mycelial growth in Δ*gcc1*, Δ*gpf1* and Δ*gta1*, and pathogenicity in Δ*gpf1*, Δ*gta1*, Δ*cnf1* and Δ*cnf2*. On the other hand, several mutant phenotypes were shared by one TF gene mutant. Also, it is fascinating to know how to regulate similar phenotypes by different TF genes. In general, every TF gene regulates the expression of many downstream genes and the expression of every gene is regulated by many TF genes, and therefore each mutant phenotype is controlled by the changes in expression of several genes. When two TF genes show similar mutant phenotype, there are at least 3 types of phenotype regulation mechanisms between them. First, TF gene A regulates another TF gene B, and TF gene B continues to regulate a group of downstream genes. Second, TF genes A and B commonly regulate a group of downstream genes. Third, TF genes A and B regulate two different groups of downstream genes independently, but they lead to similar phenotypes. However, two or three of these gene regulation types often occur simultaneously. For example, *GPF1* and other TF genes were down-regulated in Δ*cnf2*, while *CNF2* and other TF genes were also down-regulated in Δ*gpf1* (Table S11 in Text S1). A common group of genes (Figure 8) were regulated both by *CNF1* and *GPF1* which led to similar pathogenicity phenotypes in mutants, while two other different groups of genes (Figure 8) were regulated by *CNF1* and *GPF1* independently which led to different phenotypes in conidiation in mutants.

In conclusion, this study represents a major advance in a high-throughput gene knockout system suitable for filamentous fungi, provides the functional characterization of 104 Zn_2_Cys_6_ TF genes in the rice blast fungus, and reveals gene expression patterns of two virulence-required TF genes *GPF1* and *CNF2*. These studies will help us build more fungal gene-deletion mutant libraries in an economical way and uncover the transcriptional network in fungi and fungal pathogenic mechanisms.

## Materials and Methods

### Strains

*M. oryzae* strain 70-15 and its mutants, *S. cerevisiae* strain FY834, and *E. coli* strain DH5α and *A. tumefaciens* strain AGL1 were used in this study.

### Building of a yeast-*Escherichi-Agrobacterium* shuttle vector pKO1B

A 1184-bp promoter fragment of *M. oryzae H3* histone gene amplified from pKD5 [35] with primers H3sF and H3SR (Table S1 in Text S1) and 720-bp *eGFP* CDS fragment amplified from pEGFP (Clontech, USA) with primers KoGFPF and KoGFPR (Table S1 in Text S1) were inserted into the *Xho*I/*Eco*R1 sites and the *Eco*R1/*Sac*I sites of the binary vector pCAMBIA1300 (Cambia, USA) to produce the vector pKO1. pKO1B was then produced by inserting a 2.9-kb *URA3*-2micro2_origin fragment amplified from pYES2 (Invitrogen, USA) with primers uraf and urar (Table S1 in Text S1) into the *Sac*II site of pKO1 by a yeast recombinational cloning method using S. c. EasyComp Transformation Kit (Invitrogen, USA).

### Primer design and construction of gene-deletion cassettes

A list of 2-kb regions on both sides of each ORF (12775 ORF totally) was retrieved from the rice blast fungus database MG8 (www.broadinstitute.org) and saved in Excel type files by a program written by Mr. Tan Cheng. 23-nt primers (Table S1 in Text S1) of each gene-specific flank (1000 bp–1500 bp in length and 1200 bp in optimum length) were designed by the BatchPrimer3 program [76]. For each gene, primers were designed and synthesized with the following common 33-nt 5′ regions:

5f:GCTGTACAAGTAAGAGCTCGGTACCCGGGGATC… (Homologous to pKO1B)

5r:CCGGGAGATGTGGGGCACTGTGGCGTTGGCACA…(Homologous to *SUR* gene)

3f:TTGATTATTGCACGGGAATTGCATGCTCTCACA… (Homologous to *SUR* gene)

3r:TTAAGTTGGGTAACGCCAGGGTTTTCCCAGTCA… (Homologous to pKO1B)

The flank fragments were produced from genomic DNA of *M. oryzae* strain 70-15 using Primer Star or EX Taq (TaKaRa, China) in 96-well PCR plates. The *SUR* cassette fragment was generated by PCR with primers surf and surr (Table S1 in Text S1) from pBS-SUR [35]. All PCR products were verified by agarose gel electrophoresis analysis and then used in subsequent steps without further purification. Among the 163 TF genes, both flanking fragments were successfully amplified for 142 genes. However, one or sometimes both flanking fragments failed to be amplified for 21 genes even after trying several reaction conditions.

The yeast transformation procedure was conducted following a small-scale yeast transformation protocol in the pYES2 user manual (Invitrogen, USA) in 96-well deep well plates as described by Colot *et al* [29]. The cocktail mixture was made by adding 1.8 ml competent yeast cells, 100 µl linearized pKO1B by *Xba*I and *Hin*dIII (100 ng/µl), 210 µl PCR production of *SUR* cassette, 60 µl denatured salmon sperm DNA (Sangon, China) and 2.6 ml DMSO to 20.7 ml freshly prepared 1×LiAc/40%PEG-3350/1×TE solution (100 mM lithium acetate, pH 7.5; 50% PEG-3350; 10 mM Tris-HCl, pH 7.5; 1 mM EDTA). Next, 200 µl of the mixture, followed by 4 µl of 5′ and 3′ flank PCR fragments of the targeted gene, were pipetted into each well of a 96-deep-well plate with a multichannel pipette. The plates were sealed and processed further following the user manual.

After 3 days, the yeast cells cultured in SC-Ura liquid medium were collected and disrupted in Fastprep-24 homogenizer (MP, USA) at 4.0 m/s for 2 min. The plasmids were extracted with TIANprep yeast plasmid DNA kit (Tiangen Biotech, China) following the user manual. Next, the yeast plasmids were transformed into the competent cells of *E. coli* strain DH5α prepared following the Inoue method for “ultra-competent” cells [77]. Four bacterial colonies on each LB plate were placed in the wells of 96-deep-well plates, with each well containing 1 ml of LB liquid medium with 50 µg/ml kanamycin. The plates were shaken at 220 rpm overnight at 37°C.

The correctness of homologous recombinational cloning for gene-deleted cassettes was confirmed by bacterial double PCR. Common primers were designed to amplify the 5′ and 3′ flanking fragment of each target gene-deleted cassette. Forward primer KO1Bf1 for the 5′ flanking fragment and reverse primer KO1Br2 for the 3′ flanking fragment were located inside pKO1B, and reverse primer SURr1 for the 5′ flanking fragment and forward primer SURf2 for the 3′ flanking fragment were located in *SUR* cassette (Table S1 in Text S1). The resulting PCR products in knockout plasmids constructed correctly would be 1.0–1.5 kb for 5′ flanking fragment and 1.5–2.0 kb for 3′ flanking fragment. The gene deletion cassettes were further confirmed by sequencing with the primers SURf2 and SURr1. PCR and DNA sequencing results showed a success rate of 100% for the recombinant plasmids (Table S2 in Text S1). We found that it was enough to confirm the recombinant plasmids by PCR and not necessary to confirm by sequencing plasmids. The correctly built knockout plasmids were extracted from the bacterial cultures using the AxyPrep-96 plasmid purification kit (Axygen, China) following the protocol in the user manual.

### *Agrobacterium tumefaciens*-mediated transformation with knockout vectors

The knockout plasmids were transformed into the competent cells of *A. tumefaciens* strain AGL1 using the freeze/thaw shock transformation method following the procedure described elsewhere [67]. The plasmids in *Agrobacterium* cell were confirmed by culture PCR with primer set SurP1 and SurP2 (Table S1 in Text S1) for a 368-bp *SUR* gene fragment.

*M. oryzae* strain 70-15 was grown on CM medium for 12 days at 25°C under constant fluorescent light, and the conidia were harvested and transformed with the knockout plasmids mediated by *A. tumefaciens* according to a previously reported procedure [67], whereas performed in groups, usually 24 or 48 genes in a batch each time. The nitrocellulose membrane strips containing the conidia co-cultivated with *A. tumefaciens* were placed on AIM medium in the dark at 22°C for 2 days and then transferred onto the selection defined complex medium (DCM; 0.17% yeast nitrogen base without amino acids, 0.2% asparagine, 0.1% ammonium nitrate and 1% glucose, pH 6.0 with Na_2_HPO_4_) plates containing 100 µg/ml sulfonylurea, 50 µg/ml kanamycin, 400 µg/ml cefotaxime and 60 µg/ml streptomycin. Sulfonylurea-resistant transformants grown on selection medium were individually transferred onto new selection DCM plates using sterile toothpicks. A total of 8741 primary transformants corresponding to 133 Zn_2_Cys_6_ TF genes were selected on the basis of sulfonylurea resistance (Table S2 in Text S1). No transformant was obtained for the other nine genes.

### Identification of gene-deleted mutants by GFP fluorescence, double PCR and qPCR

After culture for 2 days, a little mycelium of each transformant was picked out and placed onto a glass slide. We usually placed the mycelia of six transformants on a slide in proper order. The green fluorescence emitted by transformants was then observed one by one under a fluorescence microscope. Ectopic transformants emitted green fluorescence and null mutants did not when excited under the fluorescence microscope (Figure 1D). The transformants without green fluorescence were picked out and inoculated on a new selective plate and grown for 3 days. In total, 3191 transformants without green fluorescence were screened from the primary transformants.

Extraction of genomic DNA was performed following the CTAB protocol of Rogers and Bendich [70] with modifications. A small piece (>9 mm^2^) of mycelium along with some medium in the selective plate was transferred to a 2-ml round-bottom tube, and 400 µl ddH_2_O were added along with 0.1 g porcelain beads. The mycelial cells were disrupted in a Fastprep-24 homogenizer at 4.0 m/s for 2 min; 400 µl 4×CATB buffer (4% CTAB, 100 mM Trisma base, 20 mM EDTA, 1.4 M NaCl) were then added to each tube and the tubes incubated at 65°C for 30 min. Next, genomic DNA was extracted following the normal CTAB protocol [70]. In total, genomic DNA of the 3191 transformants was extracted with the CTAB method in a high-throughput way.

The gene-deleted mutants from the transformants without green fluorescence were identified by negative screening double PCR. Double PCR was performed using primers CKF and CKR internal to the targeted gene and primers Tbl-gF and Tbl-gR for the *β-tubulin* gene (Table S1 in Text S1). PCR was performed in 25-µl reaction mixtures in 96-well PCR plates: 0.15 µl primers Tbl-gF and Tbl-gR (20 µM), 0.5 µl primers CKF and CKR (20 µM), 2.5 µl 10×PCR buffer, 0.4 µl dNTP mix (25 µM), 0.3 µl Taq (5 U/µl), 19.5 µl ddH_2_O and 1 µl genomic DNA. The PCR program was: 94°C 3 min followed by 35 cycles of 94°C for 30 s, 57°C for 30 s and 72°C for 30 s, and a final extension at 72°C for 10 min. The PCR products were detected by 1.0% agarose gel electrophoresis. If the targeted gene was deleted in a transformant, there was only one band for *β-tubulin* with 554 bp in length, appearing as a positive control; otherwise, in ectopic transformants and the wild-type strain there were two bands with one of 300–400 bp (the targeted gene) and another of 554 bp (*β-tubulin*) (Figure 1E). After checking 2280 genomic DNA samples, we identified 477 transformants with deletions in a total of 104 TF genes (Table S2 in Text S1, Figure S4A). No null mutants were found for the other 29 genes after screening all DNA samples.

For the transformants identified as null mutants in the negative screening double PCR, a second PCR was performed to verify the gene deletion event. One primer p1 or p4 (Table S1 in Text S1) was limited in the genomic DNA outside the 5′ or 3′ flanking fragment in gene-deletion cassettes, and another primer p2 or p3 (Table S1 in Text S1) was limited in the *SUR* gene in gene-deletion cassettes. If the targeted gene was deleted, there was a band with 1.2–2.0 kb in length appearing in the gel. Otherwise, there was no band for the ectopic transformant and wild-type strain 70-15 (Figure 1F). A total of 477 transformants of 104 genes identified by the preceding negative screening PCR were also identified as null mutants in this positive screening PCR (Table S2 in Text S1, Figure S4A).

The copies of transformed gene-deletion cassettes in null mutants were identified by qPCR. The concentration of DNA samples of null mutants was standardized to 25 ng/µl by DNA fluorometry. The primers for the genomic DNA of *SUR* gene are qSurF and qSurR (Table S1 in Text S1). The genomic DNA fragment coding for *β-tubulin* gene (one copy in the genome) was selected as a control. The primers of *β-tubulin* gene are qtblF and qtblR (Table S1 in Text S1). PCR mixture of 25 µl was prepared: 12.5 µl 2×RT-PCR buffer (SYBR Green, premix Ex Taq, TaKaRa), 0.5 µl forward primer and 0.5 µl reverse primer (20 µM), 2 µl genomic DNA, and 9.5 µl ddH_2_O. Real-time PCR was performed in a Mastercycler (Eppendorf, USA) with the following program: 95°C for 2 min, 40 cycles (95°C for 10 s, 60°C for 20 s), and ending with a melting curve step. Each sample was repeated three times. The copies of transformed gene-deletion cassettes in null mutants were calculated by comparing with the data of the *β-tubulin* gene and wild-type strain. The exogenous gene was inserted into the mutant's genomic DNA in an integral multiple number. If the copy number of the selective marker gene was 1.0±0.2 times the β-*tubulin* gene in genomic DNA, the mutant was regarded as containing a single insertion of the selective marker gene. As a result, 477 null mutants of 104 genes were identified as single insertion null mutants (Table S2 and Table S3 in Text S1).

The gene deletion events in mutants of ten randomly selected TF genes were reconfirmed by Southern blot (Figure S4B), which was performed according to a previously reported procedure [7].

### Complementation of null mutants with native genes

The mutants were complemented with native gene copies of the wild-type strain 70-15. First, the pKO1B-HPH was built by the replacement of P*h3*-*GFP* cassette with a *HPH* gene from pCB1003 [78] in pKO1B. The copies of the complementation genes were then cloned from the genomic DNA of the wild-type strain with the primers listed in Table S1 in Text S1 and were inserted into the *Xba*I/*Hin*dIII sites of pKO1B-HPH by the yeast recombinational cloning method. The constructed complementation plasmids were transformed into the mutants using the ATMT method, and the transformants were screened on selective medium containing 200 µg/ml hygromycin B. The gene-rescued transformants were identified by RT-PCR at the mRNA level (Figure S4C) with primers specific for the targeted genes (Table S1 in Text S1).

### Phenotypic screening analyses of knockout mutants at development stages

The phenotypic screening analyses were performed by testing 20–30 knockout mutants and one control (the wild-type strain) once in a batch according to previously reported protocols [7], [79], [80]. Mutant phenotypes were assayed in triplicate with five replicates for each strain. The values of colony growth, conidiation, conidial germination and appressorium formation of mutants in the different experimental groups were compared after normalization with the wild-type strain 70-15 in the same group.

#### Colony growth, morphology and conidiation

Mycelial blocks of 5 mm from 9-day-old wild-type strain 70-15 or mutants were inoculated in the center of CM solid medium in 6-cm plates followed by culture at 25°C under constant fluorescent light. For colony growth, the diameter of the mycelial colony was recorded and colony images were captured at 6 days post inoculation (dpi). For conidiation, the whole conidia of a 7-day-old CM-grown culture in 6-cm plates were harvested and counted using a hemacytometer.

#### Conidial germination and appressorium formation

Conidia were harvested from a 10-day-old colony growing on a CM plate by adding 0.5 ml 25 ppm Tween-20 solution to the surface of the culture. The suspension was pipetted up and down once more to ensure enough conidia for the assay. A 40-µl aliquot of spore suspension was dropped onto a sterilized plastic coverslip, which was incubated in a moist chamber at 25°C for 4 and 24 h under dark conditions. A total of 300–500 conidia were counted for conidial germination at 4 hpi and for appressorium formation at 24 hpi.

### Phenotypic screening analyses of knockout mutants under stress conditions

The phenotypes of the mutants were also screened under different stress conditions (Table S4 in Text S1) with 20–30 knockout mutants once in a batch and the wild-type strain in an experiment. Four pinpoint-like mycelia of 9-day-old strains were inoculated in the solid medium of different stress conditions in a 9-cm plate with a space interval between each other and then incubated at 25°C. Colony images were captured and the diameters of the mycelial colonies were recorded at 6 dpi. The growth rates under stress conditions were compared between strains after normalization with the wild-type strain.

The phenotypes of the mutants of 5 TF genes and their complementation strains under stress conditions were reassessed. Mycelial blocks of 5 mm from 9-day-old strains were inoculated in the center of solid medium of different stress conditions in 6-cm plates followed by culture at 25°C under constant fluorescent light, along with the strains grown on CM medium as controls. Colony images were captured and the diameters of the mycelial colonies were recorded at 8 dpi. The experiments were performed in triplicate with five replicates for each strain. Growth inhibition rate of each mutant was calculated as the growth inhibition rate = (colony diameter on CM medium−colony diameter under stress condition)÷colony diameter on CM medium×100.

### Pathogenicity tests on barley and rice

The virulence tests on barley (*Hordeum vulgare*) and rice (*Oryza sativa* cv CO39) were performed following previously reported protocols [7]. For assays with leaf explants of barley or rice, 5-mm mycelium blocks of mutants (along with the wild-type strain 70-15 and mock) were inoculated on the leaf, followed by incubation in a wet box at 25°C for 4 days. For spraying assay on rice seedlings, 4 ml conidial suspension (1×10^5^ conidia/ml) containing 0.2% (w/v) gelatin were sprayed onto 15–20 rice seedlings between the third and fourth leaf stages using an artist's airbrush. Inoculated plants were placed in a wet box at 25 C for 2 days and then allowed to grow in controlled environment chambers with a photoperiod of 12 h using fluorescent lights for 5 days.

Plant penetration by the wild-type strain or mutant appressoria was assayed on leaf explants of barley or onion cuticles according to previously reported protocols [7]. A droplet of conidia (5×10^4^ conidia/ml) was inoculated on barley leaf cuticles or onion cuticles and incubated at 25°C for 24 or 48 h. The barley leaves were then treated in methanol (overnight, room temperature) to remove chlorophyll, and fixed in alcoholic lactophenol (1 h, 95% alcohol/lactophenol = 2∶1). Appressorium penetration on barley leaf cuticle or onion cuticles was assessed under a microscope.

### RNA-sequencing analysis

The wild-type strain, Δ*gpf1* and Δ*cnf2* were grown in liquid CM medium at 25°C with shaking at 180 rpm for 2 days. The cultures were then collected and incubated in H_2_O at 25°C for 4 h. The treatments were repeated in triplicate for each strain. Total RNA was extracted from ground mycelia in liquid nitrogen with the RNeasy Plant Mini Kit (QIAGEN), and mRNA was isolated using AMPure XP beads (Beckman). RNA-seq libraries were constructed using NEBNext RNA sample preparation kit (NEB) in accordance with the standard low-throughput protocol. Samples were sequenced in a 1×100 nt way on an Illumina Hiseq2500 instrument using the TruSeq PE Cluster Kit v3 - cBot - HS (Illumina) and TruSeq SBS Kit v3-HS (Illumina). The clean reads were generated by removing adaptor sequences, tags with>10% “N”, and low quality tags by FastQC (http://www.bioinformatics.babraham.ac.uk), and were mapped to the *M. oryzae* genome database (MG8) (www.broadinstitute.org) using Tophat software [81]. The data from triple biological replicates were then analyzed using Cufflinks software and resulted in quantified genome-wide transcript levels of genes (expressed in fragments per kilobase of exon model per million mapped fragments – FPKM) [82]. The significant differences in FPKM between different samples were assayed by the Cuffdiff component of the Cufflinks package [82].

### List of genes deleted in this study

The functions of 104 Zn_2_Cys_6_ TF genes were studied by knockout and phenotypic analysis in this study (Table 1).

### Accession numbers

RNA-sequencing data were deposited in NCBI's Gene Expression Omnibus (GEO accession number GSE57146).

## Supporting Information

Figure S1 **The colonies of** ***M. oryzae*** **strains on CM medium.** The mutants of 104 Zn_2_Cys_6_ transcription factor genes and the wild-type strain 70-15 were cultured at 25°C for 6 days.
(PDF)

Figure S2 **Pathogenicity screening assay of the mutants on barley leaf explants.** The mycelial agar plugs of the mutants of 104 Zn_2_Cys_6_ transcription factor genes and the wild-type strain 70-15 were placed on intact barley leaves for 4 days.
(PDF)

Figure S3 **Pathogenicity screening assay of the mutants of 104 Zn_2_Cys_6_ transcription factor genes on rice.** (**A**) The rice seedlings were sprayed with conidial suspension (1×10^5^ spores/ml) of 101 *M. oryzae* mutants and cultured for 7 days. (**B**) The mycelial agar plugs of the mutants of 3 TF genes and the wild-type strain were placed on intact rice leaves for 4 days.
(PDF)

Figure S4 **Knockout and complementation of Zn_2_Cys_6_ transcription factor genes in** ***M. oryzae*** **.** (**A**) Knockout event of null mutants confirmed by PCR. One mutant of each TF gene (which was assayed in mutant phenotype) was selected as a representative to show the PCR identification results. The size of DNA standards are indicated on the right of lanes (M). WT, *M. oryzae* strain 70-15; **a**, bands for *β-tubulin*; **b**, bands for the targeted genes; **c**, bands for unique recombinational DNA fragments relative to the target gene-deletion event. (**B**) Null mutants of ten randomly selected TF genes confirmed by Southern blot. Genomic DNAs were digested with restriction enzymes shown in Figure S4B and separated on 0.7% agarose gels. The DNAs were individually hybridized with the probes (indicated in Figure S4B). Only one band was detected in mutants and its size was different from that in the wild-type strain, indicating that homologous recombination occurred at a single site. (**C**) Complementation of 16 Zn_2_Cys_6_ transcription factor gene-deleted mutants. The mutants were rescued with their native copy of gene in *M. oryzae* strain 70-15. RT-PCR results were shown after amplification with 35 cycles. RNA was isolated from the mycelia of the wild-type strain, mutants and complemented strains grown on CM medium. *a*, the targeted genes; *b*, *β-tubulin* gene; 1, mutants; 2, complemented strains; and 3, wild-type strain 70-15.
(PDF)

Text S1 **Supplemental tables.** **Table S1**. Primers used in this study. **Table S2**. Summary of the gene knockout of 163 Zn_2_Cys_6_ transcription factor genes in the rice blast fungus. **Table S3**. List of fungal-specific Zn_2_Cys_6_ zinc finger genes being knocked out and identification of copies of *SUR* gene in the mutants in this study. **Table S4**. Phenotypic analyses of the null mutants of 104 Zn_2_Cys_6_ transcription factor genes. **Table S5**. Comparison of phenotypes of previously characterized transcription factors between three fungal mutants (*M. oryzae*, *F. graminearum* and *N. crassa*). **Table S6**. Differentially expressed genes in Δ*gpf1* when compared with the wild-type strain (FDR<0.05). **Table S7**. Differentially expressed genes in Δ*cnf2* when compared with the wild-type strain (FDR<0.05). **Table S8**. Differentially expressed genes shared by both Δ*gpf1* and Δ*cnf2*. **Table S9**. Differentially expressed genes in Δ*cnf2* when compared with Δ*gpf1* (FDR<0.05). **Table S10**. *GPF1*- or *CNF2*-dependent genes that are dispensable for pathogenicity reported in previous studies. **Table S11**. Zn2Cys6 transcription factor genes that were listed as DEGs of Δ*gpf1* or Δ*cnf2*.
(XLSX)
