# Supplementary figures and images for: Systematic Analysis of Zn2Cys6 Transcription Factors Required for Development and Pathogenicity by High-Throughput Gene Knockout in the Rice Blast Fungus
Source: PLoS Pathog. 2014 Oct 9;10(10):e1004432. doi: 10.1371/journal.ppat.1004432 (PMC4192604; doi:10.1371/journal.ppat.1004432)

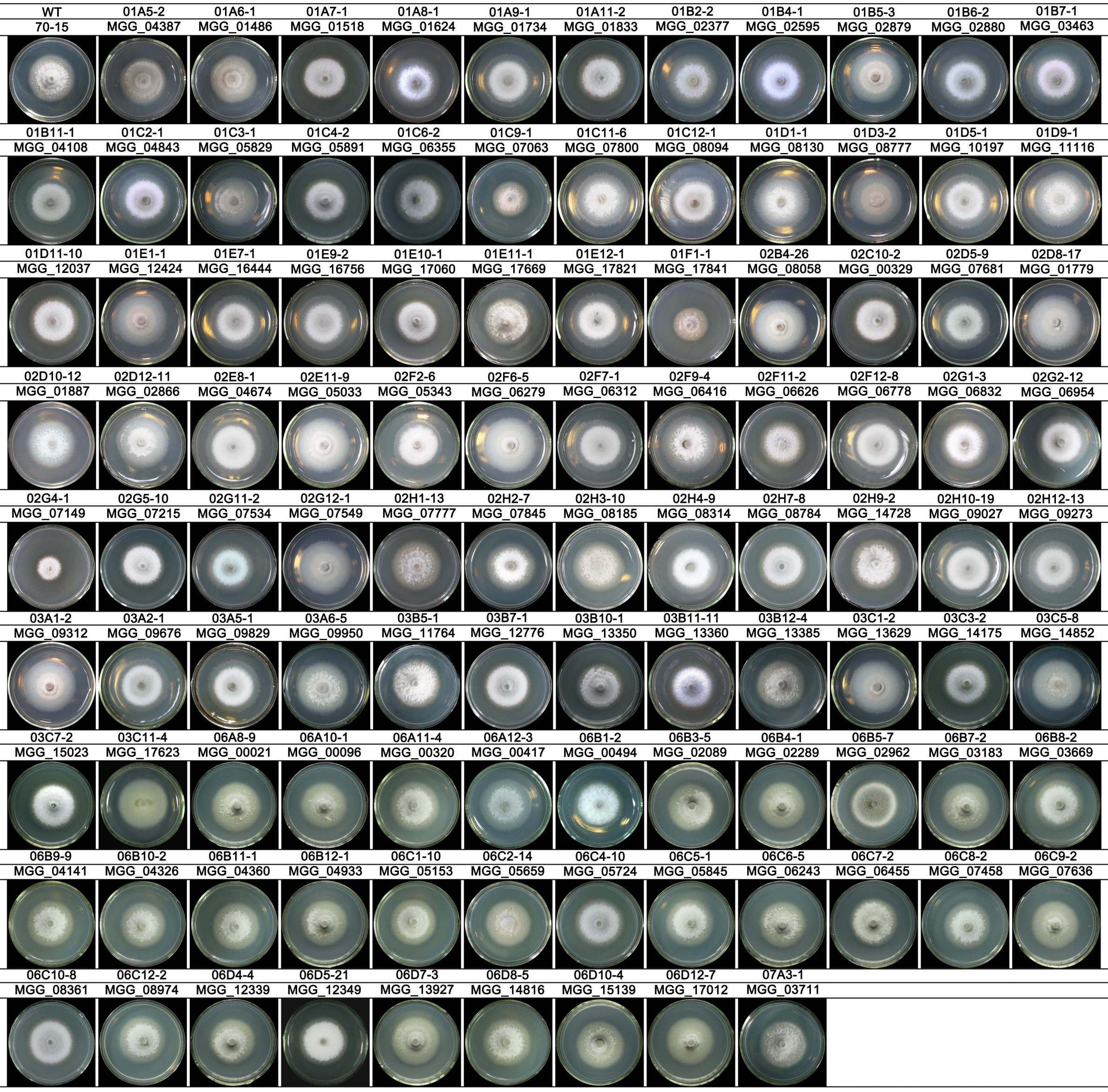

Supplement: Figure S1 — The colonies of M. oryzae strains on CM medium. The mutants of 104 Zn2Cys6 transcription factor genes and the wild-type strain 70-15 were cultured at 25°C for 6 days. (PDF) [file ppat.1004432.s001.pdf]

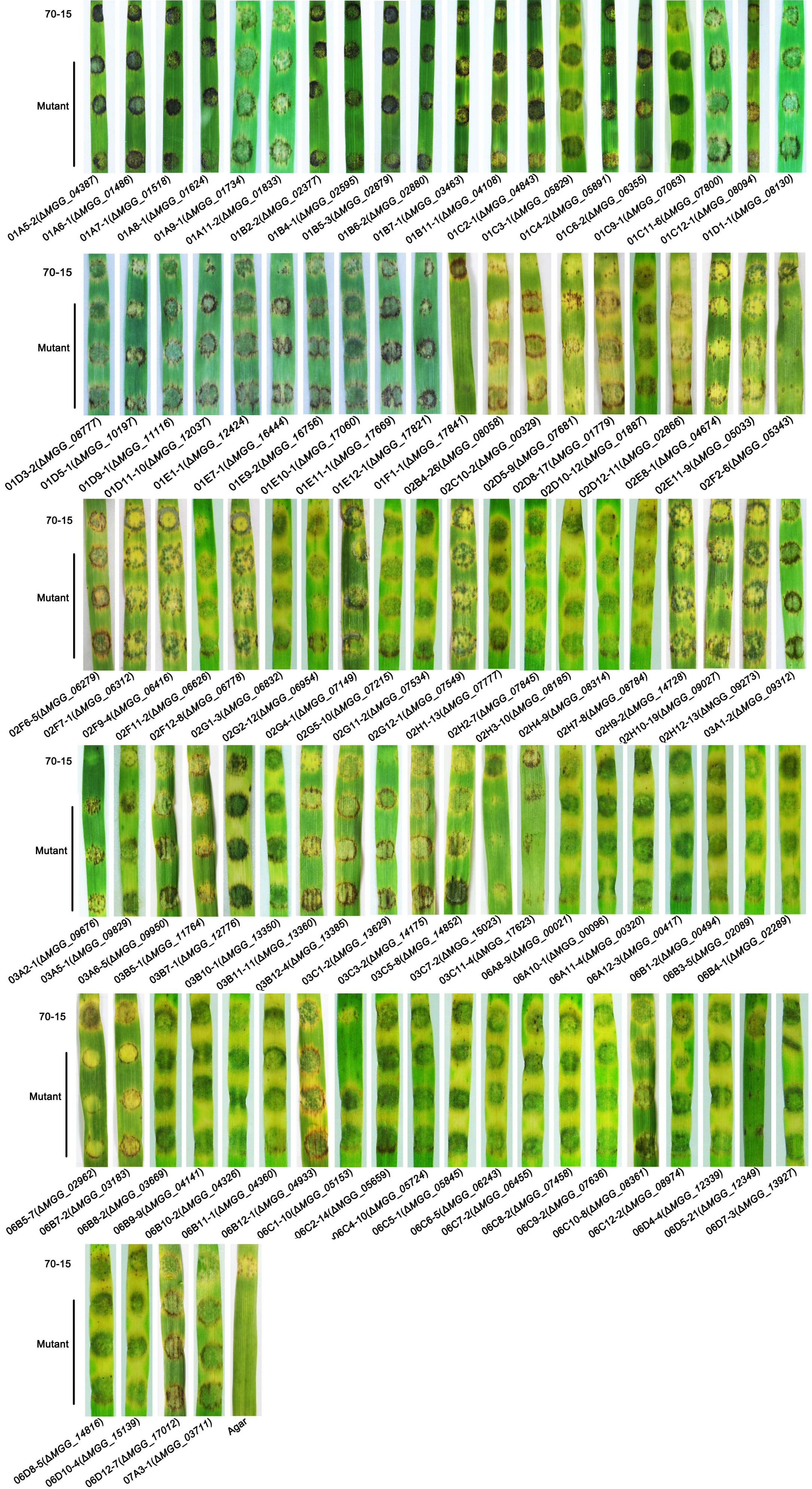

Supplement: Figure S2 — Pathogenicity screening assay of the mutants on barley leaf explants. The mycelial agar plugs of the mutants of 104 Zn2Cys6 transcription factor genes and the wild-type strain 70-15 were placed on intact barley leaves for 4 days. (PDF) [file ppat.1004432.s002.pdf]

A

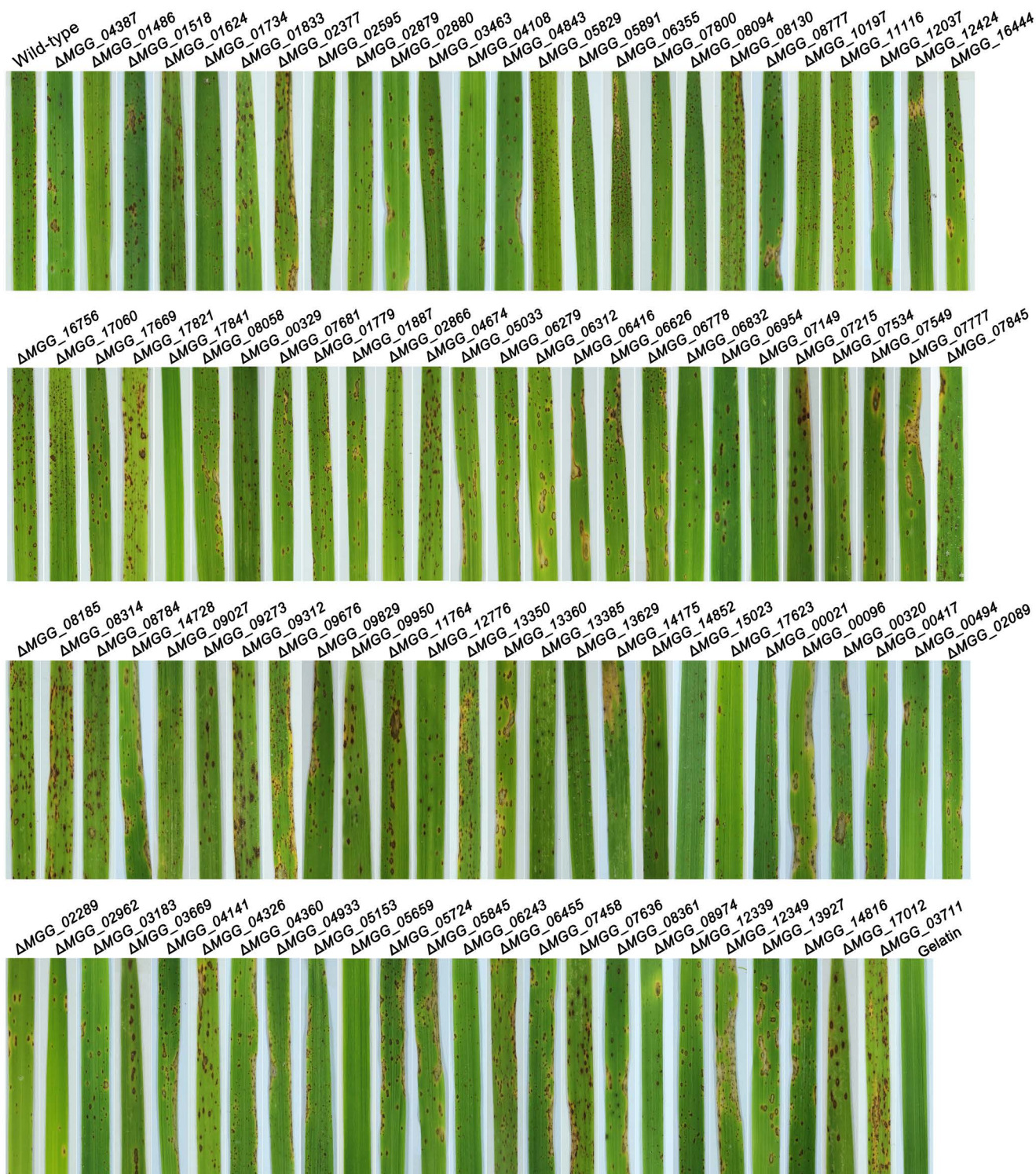

B

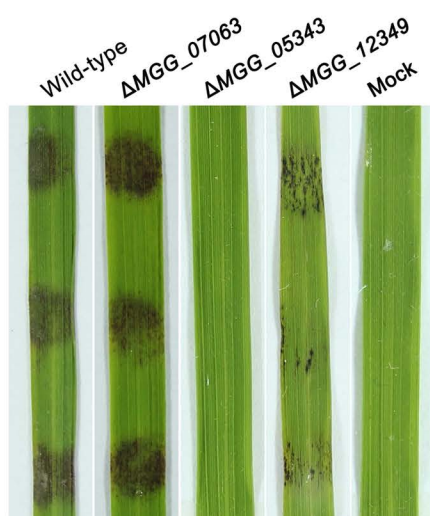

Supplement: Figure S3 — Pathogenicity screening assay of the mutants of 104 Zn2Cys6 transcription factor genes on rice. (A) The rice seedlings were sprayed with conidial suspension (1×10^5^ spores/ml) of 101 M. oryzae mutants and cultured for 7 days. (B) The mycelial agar plugs of the mutants of 3 TF genes and the wild-type strain were placed on intact rice leaves for 4 days. (PDF) [file ppat.1004432.s003.pdf]

C

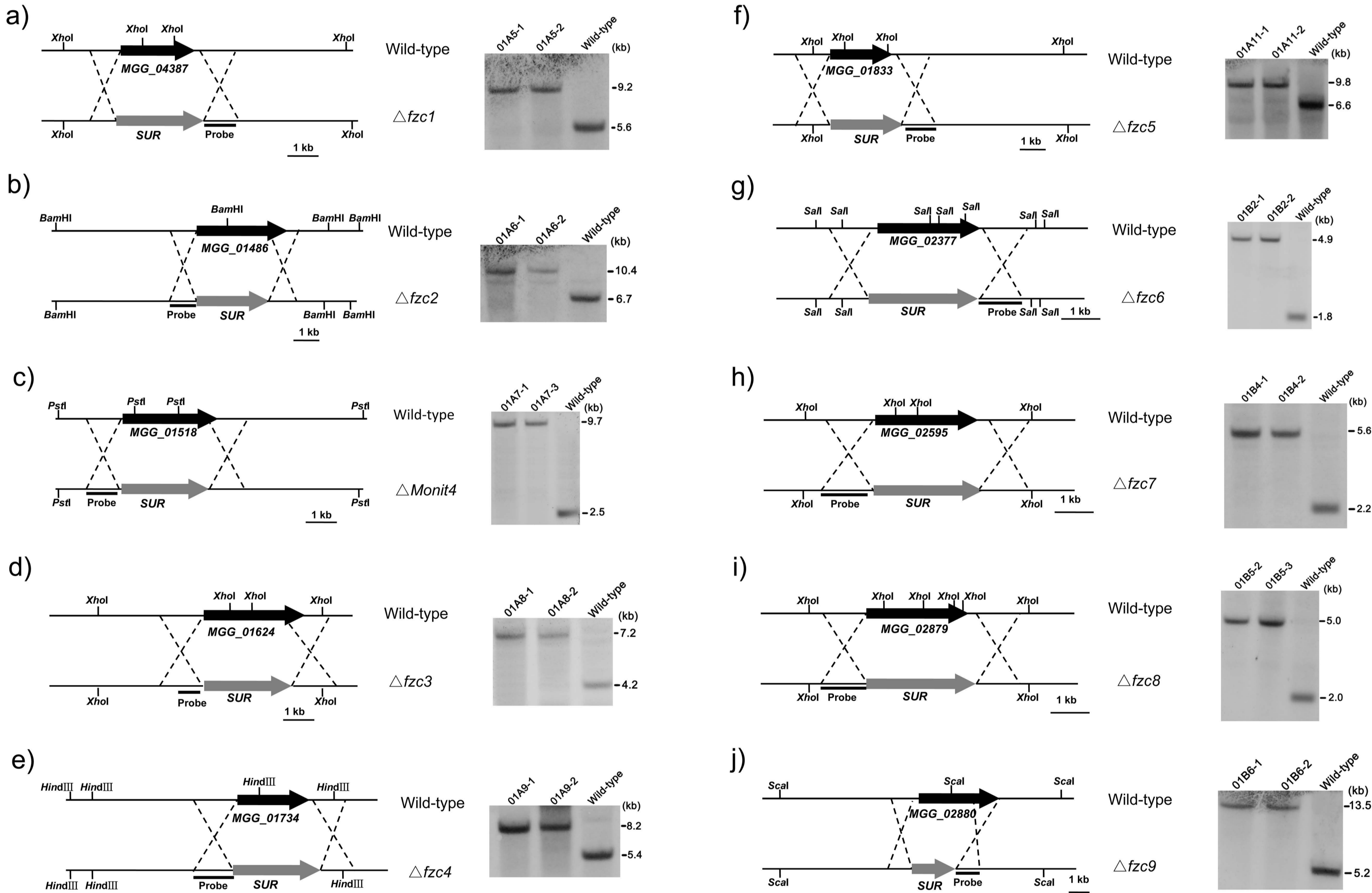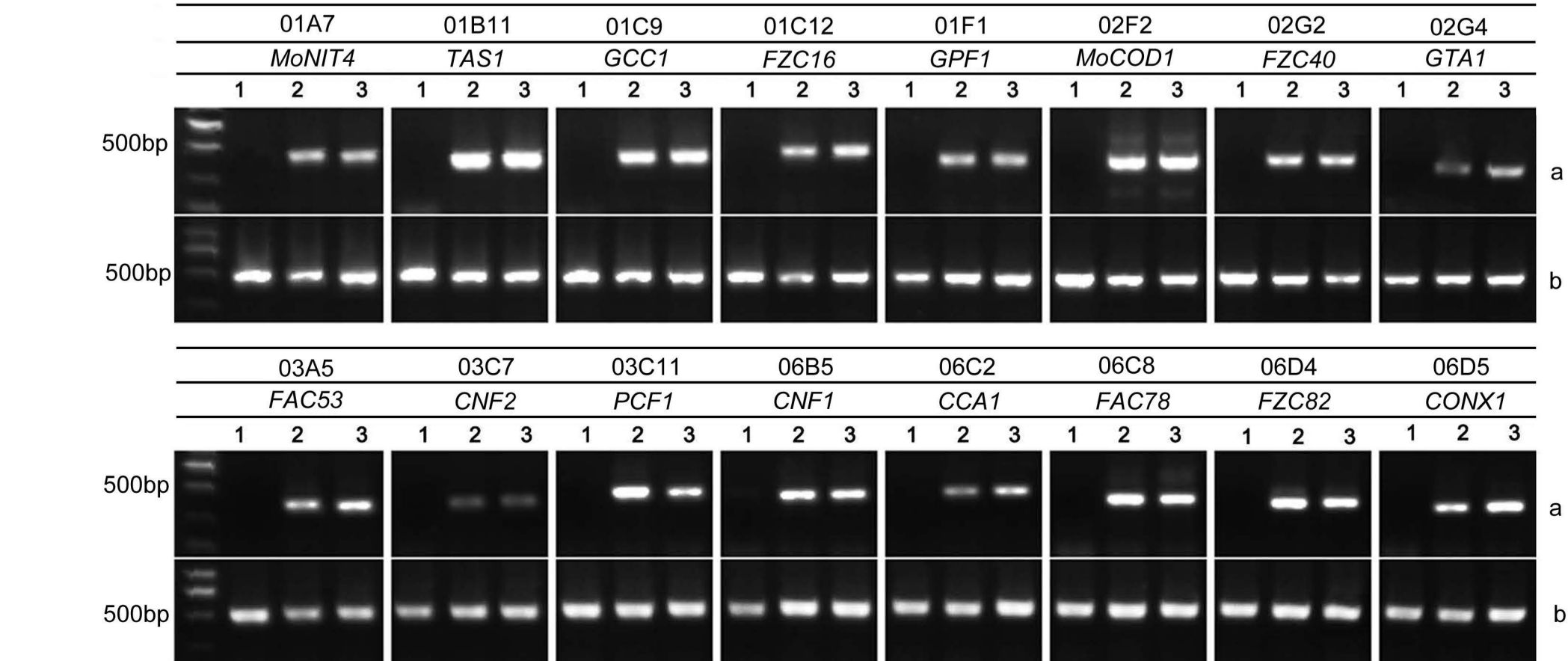

Supplement: Figure S4 — Knockout and complementation of Zn2Cys6 transcription factor genes in M. oryzae . (A) Knockout event of null mutants confirmed by PCR. One mutant of each TF gene (which was assayed in mutant phenotype) was selected as a representative to show the PCR identification results. The size of DNA standards are indicated on the right of lanes (M). WT, M. oryzae strain 70-15; a, bands for β-tubulin; b, bands for the targeted genes; c, bands for unique recombinational DNA fragments relative to the target gene-deletion event. (B) Null mutants of ten randomly selected TF genes confirmed by Southern blot. Genomic DNAs were digested with restriction enzymes shown in Figure S4B and separated on 0.7% agarose gels. The DNAs were individually hybridized with the probes (indicated in Figure S4B). Only one band was detected in mutants and its size was different from that in the wild-type strain, indicating that homologous recombination occurred at a single site. (C) Complementation of 16 Zn2Cys6 transcription factor gene-deleted mutants. The mutants were rescued with their native copy of gene in M. oryzae strain 70-15. RT-PCR results were shown after amplification with 35 cycles. RNA was isolated from the mycelia of the wild-type strain, mutants and complemented strains grown on CM medium. a, the targeted genes; b, β-tubulin gene; 1, mutants; 2, complemented strains; and 3, wild-type strain 70-15. (PDF) [file ppat.1004432.s004.pdf]
